# Abundance and phylogenetic distribution of eight key enzymes of the phosphorus biogeochemical cycle in grassland soils

**DOI:** 10.1111/1758-2229.13159

**Published:** 2023-05-10

**Authors:** Silvia Garaycochea, Nora Adriana Altier, Carolina Leoni, Andrew L. Neal, Héctor Romero

**Affiliations:** ^1^ Instituto Nacional de Investigación Agropecuaria (INIA) Estación Experimental INIA Las Brujas Canelones Uruguay; ^2^ Net‐Zero and Resilient Farming Rothamsted Research, North Wyke Okehampton UK; ^3^ Laboratorio de Organización y Evolución del Genoma/Genómica Evolutiva, Departamento de Ecología y Evolución, Facultad de Ciencias/CURE Universidad de la República Maldonado Uruguay

## Abstract

Grassland biomes provide valuable ecosystem services, including nutrient cycling. Organic phosphorus (Po) represents more than half of the total P in soils. Soil microorganisms release organic P through enzymatic processes, with alkaline phosphatases, acid phosphatases and phytases being the key P enzymes involved in the cycling of organic P. This study analysed 74 soil metagenomes from 17 different grassland biomes worldwide to evaluate the distribution and abundance of eight key P enzymes (PhoD, PhoX, PhoA, Nsap‐A, Nsap‐B, Nsap‐C, BPP and CPhy) and their relationship with environmental factors. Our analyses showed that alkaline phosphatase phoD was the dataset's most abundant P‐enzyme encoding genes, with a wide phylogenetic distribution. Followed by the acid phosphatases Nsap‐A and Nsap‐C showed similar abundance but a different distribution in their respective phylogenetic trees. Multivariate analyses revealed that pH, *T*_max_, SOC and soil moisture were associated with the abundance and diversity of all genes studied. PhoD and phoX genes strongly correlated with SOC and clay, and the phoX gene was more common in soils with low to medium SOC and neutral pH. In particular, P‐enzyme genes tended to respond in a positively correlated manner among them, suggesting a complex relationship of abundance and diversity among them.

## INTRODUCTION

Grasslands are one of the most numerous and widely distributed biomes on the Earth's surface. Factors defining grassland biomes are climatic conditions, grazing and fire (White et al., 2000; Zhou et al., 2017). They develop in arid and semi‐arid areas, with seasonal cold and dry periods, and high rates of evapotranspiration (Barnett & Facey, 2016; Knapp et al., 2002; Lenhart et al., 2015). The plant community is dominated by grasses and grass‐like species, as well as other shrubby species with different lifestyles. Plant community assemblages depend largely on climatic variables. Most of the grassland biomass above‐ground, together with the low rates of decomposition, generates significant accumulations of organic matter in soil profiles (Blair et al., 2014). Grasslands also provide several key ecosystem services, such as food, fibre and forage production, water and nutrient cycling, and erosion control. Grassland biomes are habitats for a high diversity of plants, animals and microorganisms (Blair et al., 2014; Le Roux et al., 2011).

Nutrient cycling, one of the main ecosystem services provided by grasslands, can be defined as the cycling of elements carbon (C), nitrogen (N) and phosphorus (P) between different pools (Dubeux et al., 2007). Soils have low P availability as a result of high reactivity with calcium (Ca), iron (Fe), or aluminium (Al) ions, forming insoluble complexes (Achat et al., 2016). Soil P is present in two fractions, organic (Po) and inorganic phosphates (Pi) whose proportions between soils vary depending mainly on the geological material, pH, temperature and organic matter contributions (Gaiero et al., 2020; Zhou et al., 2017). On average, the organic fraction accounts for over half of total soil P and is a valuable reservoir that could be partially mobilized by microorganisms (Condron et al., 2005; George et al., 2018; Haygarth et al., 2013). The more abundant organic P forms in soils are inositol phosphate, phospholipids, nucleic acids and teichoic acid (Condron et al., 2005; Gyaneshwar et al., 2002). Inositol phosphate (commonly called phytic acid) can account for up to 80% of total organic P (Gerke, 2015; Quiquampoix & Mousain, 2005). Phytic acid reacts with ions present in the soil forming stable and insoluble complexes and so tends to accumulate in natural grasslands soils. On the other hand, phospholipids and nucleic acids are both labile and readily accessible to soil organisms (Gerke, 2015).

The Po mineralization is strongly influenced by several factors, including soil pH, total N, precipitation and temperature, and is mediated by various enzymes with phosphatase activity. These enzymes, which are involved in different stages of the P cycle, are also influenced by such environmental factors (Margalef et al., 2017). Po‐cycle genes can be divided into three groups: Po mineralization (e.g. *phoD*, *phy*, *phoC*), transporter genes (e.g. *pstS*, *ugpQ*), and P starvation regulation genes (e.g. *phoB*, *phoR*) (Bergkemper et al., 2016; Oliverio et al., 2020; Zeng et al., 2022). The Po mineralization genes encode enzymes capable of releasing P from organic phosphate esters (henceforth P‐enzymes). The alkaline phosphatases and non‐specific acid phosphatases (Nsap) catalyse the hydrolysis between carbon and phosphorus in organic phosphate esters. The third group, the phytases, specifically release Pi from phytic acid (Bergkemper et al., 2016; Gaiero et al., 2020; Huang et al., 2009; Jorquera et al., 2008; Morrison et al., 2016; Rossolini et al., 1998). The two‐component regulatory system (PhoBR) encoded by phoBR, called the Pho regulon, regulates the transcription of P‐enzyme genes under low Pi conditions (Lidbury et al., 2017; Park et al., 2022; Santos‐Beneit et al., 2015). Alkaline phosphatases are produced by a broad range of bacteria, archaea and fungi, which play an important role in microbial P turnover (Li et al., 2021). *PhoD*, *PhoX* and *PhoA* are three different types of alkaline phosphatases, with PhoD being the most abundant and ubiquitous (Ragot et al., 2015). Both PhoD and PhoX were identified as Ca2+‐dependent extracellular enzymes and PhoA as a Zn2 + ‐dependent intracellular enzyme (Neal et al., 2018). Alkaline phosphatases show a broad substrate specificity and high catalytic efficiency (Cai et al., 2021; Rodriguez et al., 2014). These characteristics enable microorganisms harbouring these genes to use alternative P sources under P‐limited conditions, conferring them an advantage over the plants (Li et al., 2021).

Acid phosphatases are another group of enzymes distributed widely among microorganisms and plants. They are divided into three groups, Nsap class A, Nsap class B and Nsap class C, none of which exhibit strong substrate specificity, hence their names (Thaller et al., 1998). These enzymes are mostly produced by microorganisms and are mostly active in acid soils (Gaiero et al., 2018). To expand the knowledge of these enzymes, metagenomic studies have been carried out to understand how they vary in abundance and diversity in different environments (Bergkemper et al., 2016; Neal et al., 2018). Neal et al. (2018) showed that Nsap class C, a putative extracellular enzyme, was predominant in acid soils under P‐limiting conditions compared with Nsap class A a putative intracellular or periplasmic enzyme. These enzyme groups have been observed to have higher activity and gene abundance in the rhizosphere than in the bulk soil (Fraser et al., 2017; Spohn & Kuzyakov, 2013).

Phytases are produced by bacteria, fungi, plants and animals able to catalyse the mineralization of organic P from phytate to inorganic P (Ariza et al., 2013; Jorquera et al., 2008; Tu et al., 2011). Phytase families, more common in microorganisms, are the beta‐propeller phytase (BPP), protein tyrosine phosphatase‐like cysteine phytase (CPhy) and histidine acid phytase (HAPhy) (Lim et al., 2007). The main differences between the phytase families are structural, mainly related to differences in the active site which determines which phosphate group of the phytate is dephosphorylated, and co‐factor requirements. Despite this, all phytases can release the six phosphate molecules contained in the phytate (Misset, 2002). Phytases exhibit different pH and temperature optima in the laboratory (Caffaro et al., 2020) and also are dependent on the soil microorganisms species (Amadou et al., 2021). Moreover, enzymatic activity is affected by soil type, texture and mineralogy by varying the ability to retain an active enzyme (Azeem et al., 2015; Rao et al., 1994; Tang et al., 2006).

Soil microorganisms play an important role in the soil P cycle, mediating P release for plants and other living soil organisms (Awasthi et al., 2011; Richardson & Simpson, 2011). Several prokaryotic phyla have been associated with soil Po mineralization *Acidobacteria*, *Actinobacteria*, *Firmicutes* and *Proteobacteria* (Amadou et al., 2021). These Po mineralizing phyla contain a repertoire of genes that allow them to obtain Pi from organic compounds using different strategies. Forest soil study showed *Actinobacteria* and *Proteobacteria* played a dominant role in oxidative phosphorylation, whereas *Firmicutes* contributed to substrate phosphorylation (Ma et al., 2021). The alkaline phosphatase encoded by the *phoD* gene was primarily found in bacteria and was spread across 20 bacterial phyla (Ragot et al., 2015). Grassland microbiome studies showed *Actinobacteria*, *Planctomycetes* and *Proteobacteria* were the dominant bacterial phyla carrying the *phoD* gene, representing over 80% of all sequences (Graça et al., 2021). The *Streptomyces* genomes harbour alkaline phosphatases encoded by *phoA* and *phoD* genes and acid phosphatase class A coding gene (*phoC*) (Tian et al., 2021). Finally, the *Streptococcus* genus has been associated with phytase production and mineralization of phosphate (de Lacerda et al., 2016).

Grasslands are one of the five most important biomes on Earth due to the biodiversity they harbour and their economic importance. This makes it necessary to have a deeper understanding of its functions and dynamics for its preservation. This study aimed, through a global scale analysis of metagenomic data, to assess how eight key prokaryotic P‐enzymes involved in P cycling vary in their abundance and diversity in grassland biomes, how are they related between them, how they interact with the general functional profiles, and how is this related to environmental variables. We hypothesized a certain association between the P‐enzyme coding genes, and that the different soil properties and climate variables of grassland would affect the profiles of these genes. We then attempted to identify which variables could be drivers of the observed patterns.

## EXPERIMENTAL PROCEDURES

### Data collection

A total of 376 geo‐referenced metagenome samples from 17 projects deposited with MG‐RAST were selected through the TerrestrialMetagenomeDB (https://webapp.ufz.de/tmdb/) applying the following filters: Source DB: MG‐RAST; seq_technology: Ilumina; material: soil; Biome: grasslands, temperate grasslands, savanna and shrubland to assembly the grassland soil metagenomes samples set (Figure S1). All metagenomes included in the dataset were from topsoil samples (depth 10–15 cm). The set of environmental variables was assembled, including soil properties and climatic variables for each sample based on its geographic location. Soil type and physicochemical properties were obtained from SoilGrid 250 m 2.0 – ISRIC World Soil Information. The following properties were included Bulk Density (BD; cg cm^3−^‐1), Clay (g kg‐1), Sand (g kg‐1), Silt (g kg‐1), Cation Exchange Capacity at pH 7 (CEC; mmol(c) kg‐1), Total Nitrogen (N; dg kg‐1), Soil Organic Carbon (SOC; dg kg‐1), pH (water*10). The estimated organic available P (Pav) was estimated based on SOC and N content following a model proposed by Tian et al. (2010) who proposed a C:N:P ratio of 134:9:1 for organic‐rich topsoil, we estimated P content in relation to C:P and N:P ratios and took as P value the average between them. Climate variables were obtained from TerraClimate (https://www.climatologylab.org/terraclimate.html), including maximum temperature, (*T*_max_;°C), Precipitation (ppt; mm), actual evapotranspiration (aet; mm), soil moisture (moisture; mm) and runoff (q; mm) (Table S1). Hereafter they are called environment variables. The collinearity analysis on the environmental variables set was performed with R‐base (R core Team 2022), We included variables with *r* ≤ 0.5 and meaningful to the study.

The functional annotation based on MG‐RAST subsystems level 2 of the 376 selected metagenomes (Table S2) was obtained from the MG‐RAST repository (Meyer et al. 2008).

The set of predicted proteins in each metagenome was obtained through the RESTful API of MG‐RAST (Wilke et al., 2015). Protein sequences were downloaded using a matR version 0.9.1 package R (Braithwaite & Keegan, 2018).

The set of 376 samples showed imbalances because of the overrepresentation of the same sites, particularly from the northern hemisphere (much more studied) compared with the southern hemisphere. To minimize this bias, subsequent analyses were performed on a balanced reduced subset of 74 grasslands soil metagenomes. This subset included a maximum of three samples per MG‐RAST project with the same geo‐reference. In addition, soil metagenome data from two Uruguayan sites were generated for this study (Table S3). In the subset, we excluded the samples under high‐impact treatments (e.g. fertilization, tillage, etc.). All analyses were performed on this reduced subset of 74 samples from 17 MG‐RAST projects (Table S3).

Soil metagenomic sequencing from Uruguay (projects mgp91922 and mgp93346) was carried out on a HiSeq Illumina platform, (Service CD Genomics, NY; pair‐end read 150 bp). Raw sequence quality was analysed with FastQC software version 0.11.2. Assembly and functional annotation were performed on the MG‐RAST repository. Raw sequence data are publicly available on the MG‐RAST repository. Functional annotation based on MG‐RAST subsystems level 2 of the 376 selected metagenomes was obtained from the MG‐RAST repository (Meyer et al. 2008). The set of predicted proteins of each metagenome was obtained through the RESTful API of MG‐RAST (Wilke et al., 2015). Protein sequences were downloaded using matR version 0.9.1 package R (Braithwaite & Keegan, 2018).

### P‐enzyme gene identification and phylogenetic analyses

The reference databases of the P‐enzyme used in this work were built by Neal et al. (2017). The P‐enzymes included are listed in Table 1. It is important to note that the use of any reference database introduces a certain bias in the search space.

**TABLE 1:** List of P‐enzymes included in the analyses.

P‐enzyme	Gene	Predicted cellular localization	Number of protein sequences in the reference database
PhoA	*phoA*	Periplasmic/Cytoplasmic	293
PhoD	*phoD*	Outer membrane/extracellular	833
PhoX	*phoX*	Outer membrane/extracellular	424
Nsap class A (Nsap‐A)	*phoC*	Periplasmic/Cytoplasmic	750
Nsap class B (Nsap‐B)	*aphA*	Periplasmic/Cytoplasmic	388
Nsap class C (Nsap‐C)	*olpA*	Outer membrane/extracellular	1123
β‐propeller phytase (BPP)	*phyL, phyS*	Outer membrane/extracellular	108
Cysteine phytase (Cphy)	*phyA*	Outer membrane/extracellular	122

Protein sequence alignments of the respective reference database were performed using MAFFT version 7.4.60 (Katoh et al., 2002) under default parameters. Reference protein phylograms were inferred with IQTree 2 version 1.6.12 (Minh et al., 2020) and the evolutionary models were evaluated with RAxML‐NG (Kozlov et al., 2019). Phylograms were plotted with iTOL (Interactive Tree of Life; Letunic & Bork, 2007).

To determine the abundance and diversity of the P‐enzymes in the metagenomes, we queried each metagenomic sample against each P‐enzyme reference database. First, the whole predicted protein set of each metagenomic sample was queried against each P‐enzyme reference database using HMMER version 3.3.1 (http://hmmer.org) keeping hits with an e‐value below 1e‐5. Then, these sequences were aligned to the correspondent reference database alignment using MAFFT—‐add sequence option and default parameters.

Maximum likelihood‐based phylogenetic placement of metagenome‐derived protein sequences on the appropriate P‐enzyme reference phylogenetic tree was performed with EPA‐ng (Barbera et al., 2019). Edge‐PCA ordination and Kantorovich‐Rubinstein (KR) distance metrics (Evans & Matsen, 2012; Matsen & Evans, 2013) were computed on these results. The edge‐PCA and KR distances were performed using gappa (Czech et al., 2020), and tree and domain composition diagrams were drawn using Archaeopteryx (https://sites.google.com/site/cmzmasek/home/software/forester).

### Statistical analyses

Canonical analysis of principal coordinates (CAP) (Anderson & Willis, 2003) implemented in Vegan R Package version 2.6.2 (Oksanen et al., 2019) was performed based upon Mahalanobis distance to calculate the relationship between the metagenomes functional profiles (subsystems level 2) and environmental variables. The significance of the model parameters was determined with permutational multivariate analysis of variance (PERMANOVA) with 999 permutations.

The protein/function count matrix (level 4 in the MG‐RAST nomenclature), including the eight P‐enzymes, for the 74 selected metagenomes was normalized with CPM and TMM methods using the edgeR package (Robinson et al., 2010). This data was used to perform the direct correlations of P‐enzymes with environmental variables.

Canonical analysis of principal coordinates (CAP) (Anderson & Willis, 2003) implemented in the Vegan R Package version 2.6.2 (Oksanen et al., 2019) was used to evaluate the relationship between the abundance and diversity of P‐specific functions with the environmental variables. CAP analysis associating P‐enzyme abundance with environmental variables was performed using Mahalanobis distance. When appropriate, each P‐enzyme abundance in each sample was normalized in relation to the sequencing coverage of each P‐enzyme. The significance of the model parameters was determined with permutational multivariate analysis of variance (PERMANOVA) with 999 permutations.

The KR distance of each P‐enzyme calculated as mentioned above was used to perform the distance‐based CAP analyses between the abundance and diversity of each P‐enzyme and environmental variables. The significance of the model parameters was also determined with PERMANOVA based on 999 permutations. Graphics were produced with the R package ggplot2 (Wickham, 2016). All basic statistical procedures were performed using R‐base (R core Team 2022). All taxonomy names cited are mentioned in italics and agree with Thines et al., 2020.

## RESULTS

### Metagenome functional profiles and environmental variables

First, we wanted to generate a general perspective of grassland functional landscapes and their relationship with environmental variables. To this aim, we performed a Canonical Analysis of Principal Coordinates (CAP) on a set of 74 grassland soil metagenomes (a reduced data set to correct for imbalances in the sample number per site, see methods). We used as input 168 functional processes (level 2 of the subsystems annotation from MG‐RAST, Table S4) and their corresponding environmental variables (Table S3). The constrained model was significant (*p* = 0.001) and explained 24.8% of the total variance observed in the data set. Significant associations (*r* > |0.20|, *p* < 0.01) between the distribution of metagenomes and nine environmental variables were identified. CAP1 axis was correlated with pH (*r* = −0.743), bulk density (BD, *r* = −0.521), soil organic carbon (SOC, *r* = 0.564) and soil moisture (*r* = 0.536). This axis separated samples from low pH soils with average values of 5.65 (e.g. mgp9904, mgp5588, mgp91922 and mgp93346) from those with neutral pH (mgp13948 among others). CAP2 axis was mainly associated with pH (*r* = −0.486), SOC (*r* = −0.220), *T*_max_ (*r* = 0.664), runoff (q, *r* = 0.490), soil moisture (*r* = 0.476) and Clay (*r* = 0.231). Extreme values of the CAP2 axis corresponded to mgp10450 and mgp10451 (both from Brazil) which were associated with the highest *T*_max_ (26°C), soil moisture (115.5 mm) and precipitation (ppt) (129 mm) values of the set (Table 3A and Figure S2). To validate the subsampling (74 vs. 376 sample set), we performed CAP analysis in the larger set and examined the correlation between the axes of both analyses. We observed a high positive correlation between the correspondent first and second axes (correlation values >0.60).

### Analyses on the abundance of P‐enzymes coding genes

**FIGURE 1:**
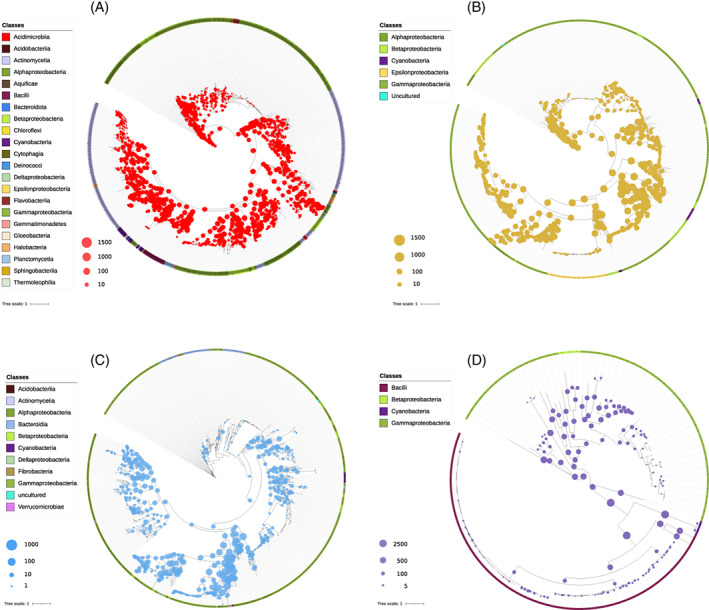
Phylogenetic placements of the predicted proteins of each metagenome with respect to the reference bases of each enzyme: (A) PhoD, (B) PhoX, (C) Nsap‐A and (D) BPP. The size of the circle representing placements is proportional to the abundance. Maximum likelihood‐based phylogenetic placement of metagenome‐derived protein sequences was performed with EPA‐ng and a tree was drawn with iTOL. The circle sizes represent the number of hits per node. The outer circle shows bacterial classes included in the reference trees.

We interrogated the predicted protein set of each metagenome against the reference database of PhoD, PhoX, PhoA, Nsap‐A, Nsap‐B, Nsap‐C, BPP and CPhy enzymes to obtain the abundance and phylogenetic distribution of P‐enzyme coding genes. Inferred protein relative abundance in each soil metagenome is shown in Table S7 and phylogenetic placements are in Figures 1 and S3.

The alkaline phosphatase genes were the most abundant in the dataset, eight times higher than the acid phosphatase genes and 58 times higher than the phytase genes, independently of the soil properties (Table 2). We also observed differences in the abundance and phylogenetic distribution within each group of P‐enzyme genes. The alkaline phosphatase gene *phoD* showed an abundance of five times higher compared with *phoX* and 20 times higher compared with *phoA*. Both genes, *phoD*, and *phoX* had broad phylogenetic distributions and no clear dominant phylotypes (Figures 1 and S3), contrary to the limited phylogenetic distribution observed in *phoA* (Figure S3 and Table S5).

**TABLE 2:** Median relative abundance of each P‐enzyme.

P‐enzyme	PhoD	PhoX	PhoA	Nsap‐A	Nsap‐B	Nsap‐C	BPP	Cphy
Gene	*phoD*	*phoX*	*phoA*	*phoC*	*aphA*	*olpA*	*phyL, phyS*	*phyA*
Median relative abundance (No. of hits)	468.6	96.8	23.3	29.7	0.55	39.9	10.3	0

Genes encoding Nsap‐A and Nsap‐C were the most abundant of the acid phosphatases, with similar abundances (Nsap‐C coding gene was 1.3 times higher than Nsap‐A one) (Table 2, Table S5), but a different distribution in their corresponding phylogenetic trees. Whilst Nsap‐A coding gene showed a broad distribution within its phylogeny (Figure 1c), Nsap‐C one was concentrated in the main branches of *Gammaproteobacteria*, *Flavobacteria* and *Sphingobacteria* classes (Figure S3). On the other side, Nsap‐B had a low abundance and only *Gammaproteobacteria* variants were found (Figure S3 and Table S5).

BPP coding gene (*phyL* and *phyS*) was the most abundant of the phytases genes and presented a phylogenetic distribution mainly restricted to the *Proteobacteria* phylum (e.g. *Pseudomonas*, *Alteromonas* and *Acinetobacter*) (Figure 1d and Table 2). The CPhy coding gene (*phyA*), with lower abundance, was distributed within *Betaproteobacteria*, *Gammaproteobacteria* and some classes of the *Firmicutes* phylum (Figure S3 and Table 2).

First, we performed simple correlation analyses between normalized genes encoding P‐enzyme abundance (by CPM and TMM methods, obtaining equivalent results) and environmental variables showed that *phoD*, *phoX* and *phyL* and *phyS* (BPP) coding gene had a significant correlation (*p* < 0.001) with pH, actual evapotranspiration (aet), precipitation (ppt), runoff (q) and soil moisture. In addition, we observed that *phoD* showed significant correlations (*p* < 0.001) with SOC and estimated organic available P (Pav). Nsap‐C coding gene (*olpA*) showed a significant correlation with aet, *q*, ppt and moisture (Table S6). We then move forward to multivariate analyses.

We used CAP analysis to explore the relationship between P‐enzyme coding genes normalized abundance and environmental variables (Figure S4). The constrained model based on Mahalanobis distance explained 36.4% of the variance within the data set (*p* = 0.001). We identified the alkaline phosphatase genes *phoD*, *phoX* and *phoA* were mainly responsible for the explained variance. CAP1 axis explained 8.9% of the variance (*p* = 0.001) and was associated with pH (*r* = −0.70), BD (*r* = −0.48), Sand (*r* = −0.40), ppt (*r* = 0.74), aet (*r* = 0.67), SOC (*r* = 0.52), Pav (*r* = 0.52) and Silt (*r* = 0.49). We observed that this axis separated samples from metagenomes of clay soils with low pH (5.0–6.8) and high SOC values (Androsols, Cambisols, Ferrasols, Fluvisols, Kastanozem/Luvisol, Luvisol/Kastanozem) from those of neutral or alkaline soils, having lower SOC contents (Chernozem, Luvisol and Kastanozem). CAP2 axis was associated with *T*_max_ (*r* = −0.40), BD (*r* = −0.34), pH (*r* = −0.27), ppt (*r* = 0.41), actual evapotranspiration (aet, *r* = 0.39) and runoff (q, *r* = 0.39). This axis separated soil metagenomes associated with lower aet values and relatively high *T*_max_. All variables were significant with a *p* < 0.001 (Table 3A).

**TABLE 3:** CAP analyses based on Mahalanobis distance for MG‐RAST Functional profiles annotations (level 2 Subsystem) and P‐Enzyme relative abundance (A) and based on Kantorovich‐Rubinstein distance for each of eight enzymes (B)

(A)																		
Variables	Subsystems			Relative abundance P‐enzymes			Variables			Subsystems			Relative abundance P‐enzymes			Variables		
	*p* value			CAP1 (*r*)						*p* value			CAP1 (*r*)					
pH	0.001			−0.74			−0.49			0.001			−0.70			−0.27		
SOC	0.001			0.56			−0.22			0.009			0.52			0.21		
Pav	0.059			0.57			−0.29			0.004			0.52			0.12		
CEC	0.057			0.15			−0.16			0.086			0.28			0.04		
BD	0.001			−0.52			−0.12			0.001			−0.48			−0.34		
Clay	0.001			−0.04			0.23			0.003			0.12			0.18		
Sand	0.001			0.12			0.07			0.001			−0.4			−0.02		
Silt	0.070			−0.14			−0.31			0.001			0.49			−0.12		
aet	0.001			0.65			0.18			0.030			0.67			0.38		
*q*	0.001			0.57			0.49			0.002			0.72			0.39		
Mositure	0.002			0.54			0.48			0.079			0.82			0.12		
(B)																		
Variables	Nsap—A			Nsap—B			Nsap—C			PhoA			PhoD			PhoX		
	*p* value	CAP1 (*r*)	CAP2 (*r*)	*p* value	CAP1 (*r*)	CAP2 (*r*)	*p* value	CAP1 (*r*)	CAP2 (*r*)	*p* value	CAP1 (*r*)	CAP2 (*r*)	*p* value	CAP1 (*r*)	CAP2 (*r*)	*p* value	CAP1 (*r*)	CAP2 (*r*)
pH	0.028	0.06	0.27	0.120	0.47	−0.28	0.012	0.14	0.12	0.074	−0.04	−0.29	0.001	−0.75	0.06	0.008	0.12	−0.08
SOC	0.045	−0.11	−0.18	0.523	−0.64	−0.19	0.016	0.02	−0.26	0.100	−0.18	0.12	0.001	0.22	−0.10	0.007	−0.05	−0.37
Pav	0.197	−0.18	−0.17	0.186	−0.60	−0.27	0.106	−0.02	−0.28	0.325	0.11	−0.35	0.057	0.18	−0.15	0.038	−0.08	0.00
CEC	0.001	−0.38	0.23	0.907	−0.43	−0.07	0.158	0.02	−0.01	0.015	0.05	0.45	0.001	−0.22	−0.17	0.041	−0.08	−0.25
BD	0.026	0.09	0.13	0.002	0.65	0.16	0.034	0.07	0.43	0.433	0.43	−0.05	0.001	−0.39	0.16	0.074	0.03	0.18
Clay	0.061	0.00	−0.35	0.649	−0.30	0.140	0.001	0.18	−0.69	0.070	−0.17	−0.040	0.002	0.16	−0.02	0.010	0.27	0.02
Sand	0.002	0.17	0.39	0.027	0.40	−0.05	0.033	−0.02	0.53	0.571	−0.35	0.38	0.001	−0.28	0.16	0.017	−0.09	−0.05
Silt	0.004	−0.34	−0.26	0.467	−0.34	−0.06	0.101	−0.16	−0.13	0.460	−0.02	0.39	0.001	0.27	−0.22	0.142	−0.10	0.06
aet	0.001	0.01	−0.43	0.848	−0.59	0.32	0.001	0.17	−0.21	0.015	0.11	0.46	0.001	0.78	0.01	0.001	0.28	−0.11
*q*	0.050	−0.01	−0.46	0.002	−0.80	0.27	0.023	0.00	−0.36	0.582	0.16	0.40	0.050	0.72	−0.07	0.055	0.00	−0.46
mositure	0.071	−0.27	−0.51	0.158	−0.68	0.19	0.044	−0.17	−0.45	0.580	−0.10	0.10	0.033	0.77	−0.20	0.085	−0.18	−0.12
ppt	0.082	0.00	−0.48	0.405	−0.75	0.32	0.009	0.09	−0.30	0.458	0.15	0.45	0.092	0.80	−0.03	0.146	0.16	−0.31
*T*_max_	0.001	−0.24	−0.17	0.508	0.09	0.31	0.001	−0.50	−0.04	0.057	−0.18	−0.51	0.001	0.31	−0.41	0.001	−0.61	0.04
	*p* value	% variance		*p* value	% variance		*p* value	% variance		*p* value	% variance		*p* value	% variance		*p* value	% variance	
CAP model	0.001	33.70		0.050	32.60		0.001	35.70		0.001	13.00		0.001	49.80		0.001	41.40	
CAP 1 axis	0.001	11.54		0.001	11.60		0.001	13.90		0.030	8.70		0.001	19.80		0.001	19.00	
CAP 2 axis	0.01	4.20		0.890	4.10		0.009	4.70		0.570	3.90		0.001	12.90		0.05	4.70	
Variables			BPP								Cphy							
			*p* value			CAP1 (*r*)			CAP2 (*r*)		*p* value			CAP1 (*r*)		CAP2 (*r*)		
pH			0.001			0.440			0.060		0.010			0.570		0.290		
SOC			0.066			−0.280			−0.480		0.193			−0.260		−0.180		
Pav			0.048			−0.330			−0.530		0.007			−0.340		−0.160		
CEC			0.007			−0.370			−0.620		0.010			−0.320		−0.420		
BD			0.001			0.270			0.360		0.122			0.360		0.100		
Clay			0.022			−0.090			0.050		0.526			0.190		−0.040		
Sand			0.001			0.280			0.260		0.222			−0.110		0.130		
Silt			0.004			−0.350			−0.470		0.302			0.030		−0.160		
aet			0.012			−0.310			−0.370		0.928			−0.490		−0.470		
q			0.01			−0.370			0.060		0.002			−0.330		−0.380		
Mositure			0.169			−0.620			−0.230		0.030			−0.680		−0.400		
ppt			0.164			−0.360			−0.190		0.420			−0.440		−0.460		
*T*_max_			0.001			−0.290			0.770		0.508			0.150		0.000		
			*p* value			% variance					*p* value			% variance				
CAP model			0.001			47.000					0.030			49.500				
CAP 1 axis			0.001			23.000					0.010			10.700				
CAP 2 axis			0.001			5.000					0.100			7.6000				

*Note*: Environmental variables included: pH, Soil Organic Carbon (SOC), Estimated organic available P (Pav), Cation Exchange Capacity (CEC), Bulk Density (BD), Clay, Sand, silt, actual evapotranspiration (aet), runoff (*q*), soil moisture (moisture), precipitation (ppt) and maximum Temperature (*T*_max_) (Table 3B). PERMANOVA analysis with 999 permutations was performed to determine the significance between the sites/MG‐RAST project. The values marked with red are significant at *p* < 0.01.

### Analyses on abundance and phylogeny of P‐enzyme coding genes

To gain deeper insight into the diversity and abundance of P‐enzymes coding genes, we performed CAP analyses using Kantorovich–Rubinstein (KR‐CAP) distance matrices between samples to include not only abundance but also phylogenetic information.

**FIGURE 2:**
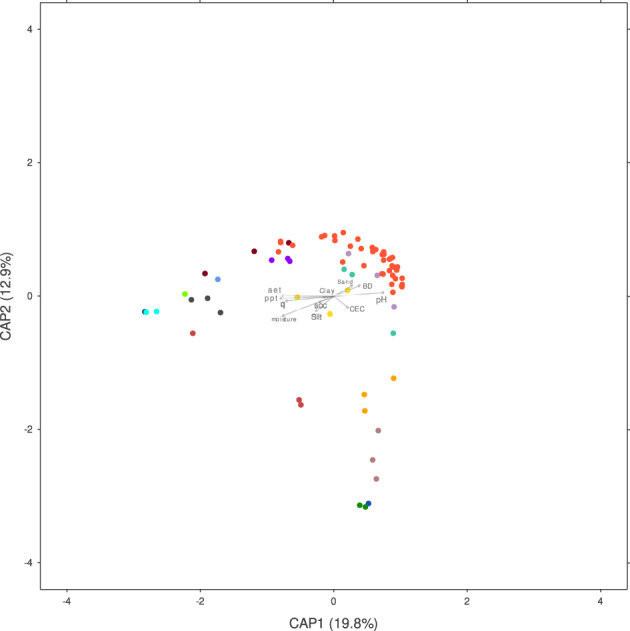
CAP based on Kantorovich‐Rubinstein distance for *phoD*. PERMANOVA analysis with 999 permutations was performed to determine the significance between the sites/MG‐RAST project. For each MG‐RAST project, three samples with the same geo‐reference were included. Each point represents samples from the project mpg1992 (blue); mpg3520 (green); mpg5588 (dark red); mpg7792 (grey); mpg8624 (mustard); mgp9904 (violet); mgp10450 (dark blue); mgp10523 (stone blue); mgp10541 (turquoise); mgp10956 (yellow); mgp13011 (lilac); mgp13520 (jade); mpg13948(orange); mpg20922 (brown); mgp89409 (brick‐red); mgp91922. (light green); mgp93346 (light blue). Vector lengths represent the correlation between each variable and the axes. CAP analysis was performed with the Vegan R package and graphics were produced with the R package ggplot2.

The *phoD* KR‐CAP analysis explained 49.8% of the total variance in the data set (*p* < 0.001). Eleven out of 13 environmental variables were associated with the first two KR‐CAP axes. The KR‐CAP1 axis was negatively associated with pH (*r* = −0.75), BD (*r* = −0.39), Sand (*r* = −0.28), CEC (*r* = −0.22) and positively with ppt (*r* = 0.80), aet (*r* = 0.79), q (*r* = 0.72), *T*_max_ (*r* = 0.313), Silt (*r* = 0.27) and SOC (*r* = 0.22). This axis separated soils with low pH, relative high values of SOC and *T*_max_ (Cambisols, Ferrasols, Mollisols/Phazoem, Luvisol/Kastanozem) from soils with higher pH and lower *T*_max_. The KR‐CAP2 axis was characterized by a negative association with *T*_max_ (*r* = −0.41), soil moisture (*r* = −0.29) and Silt (*r* = −0.22). This axis separated soil with neutral pH and relatively high Silt and Sand values from the rest of the samples (Table 3B and Figure 2).

We performed the same analysis for the rest of the P‐enzymes coding genes and the results are summarized in Table 3B (Figures [Link], [Link]). Notably, pH, *T*_max_ and aet were associated with all P‐enzyme coding gene distributions. SOC displayed a high correlation with alkaline phosphatases *phoD* and *phoX*, and acid phosphatases Nsap‐A and Nsap‐C coding genes, and estimated organic available P (Pav) was mainly associated with the phytases coding genes. Next, CEC showed a high correlation with alkaline phosphatases and phytase coding genes. Finally, clay content was related mainly to alkaline phosphatase coding genes (Table 3B).

### Covariation of P‐enzymes genes

**FIGURE 3:**
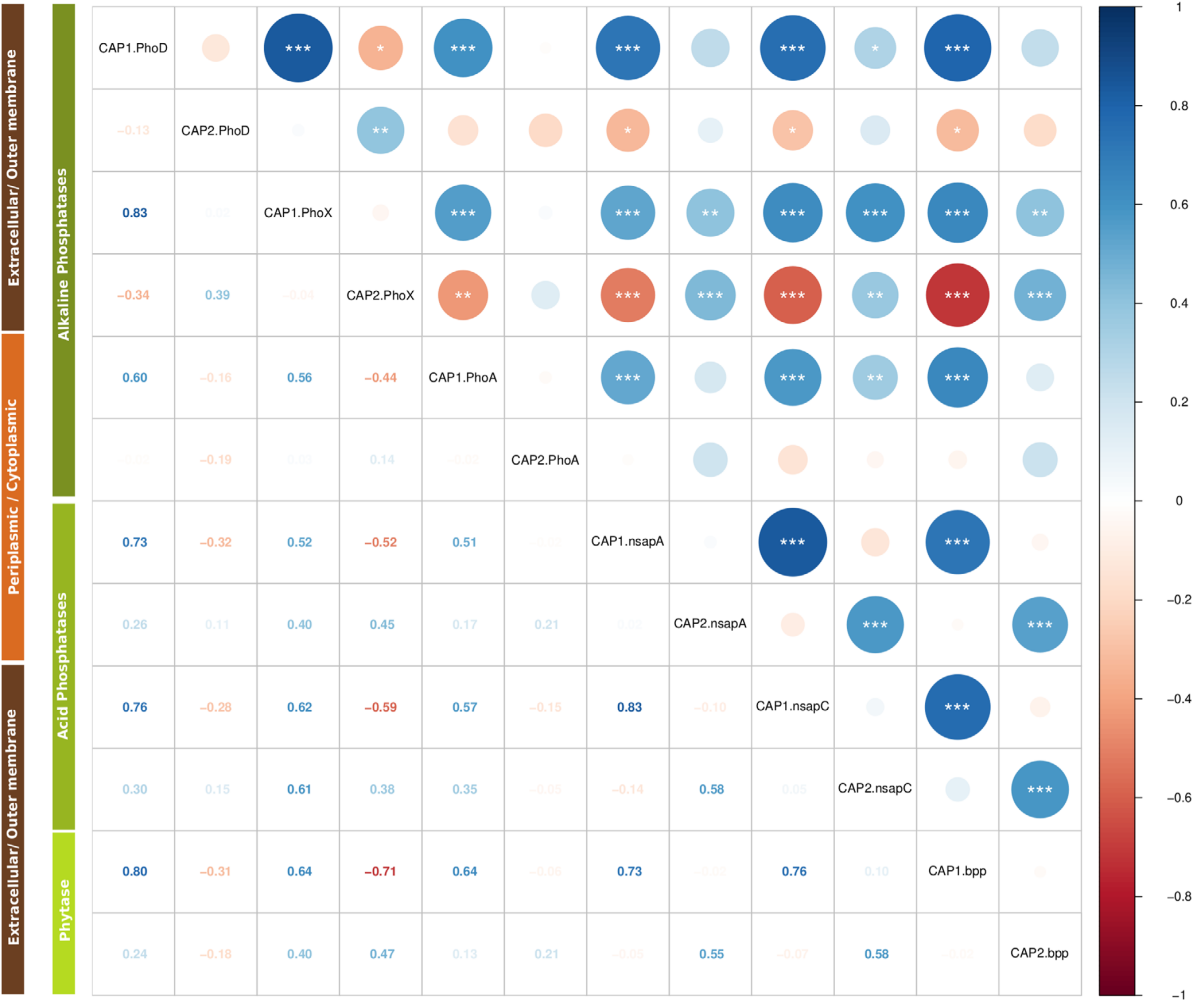
Correlation matrix of KR‐CAP axes. (A) Correlogram of the alkaline phosphatases displays the Pearson correlation coefficients between KR‐CAP PhoD axes, KR‐CAP PhoX, and KR‐CAP PhoA; KR‐CAP PhoX and KR‐CAP PhoA. The correlation coefficients are coloured according to their values; blue is the positive values and red is the negative values. (B) Correlogram of the acid phosphatases displays the Pearson correlation coefficients between KR‐CAP Nsap‐A axes, KR‐CAP Nsap‐B and KR‐CAP Nsap‐C; KR‐CAP Nsap‐B and KR‐CAP Nsap‐C. The correlation coefficients are coloured according to their values; blue is the positive values and red is the negative values. (C) Correlogram of the phytases displays the Pearson correlation coefficients between KR‐CAP BPP axes and KR‐CAP CPhy. The correlation coefficients are coloured according to their values; blue is the positive values and red is the negative values. (D) Correlogram of the most abundance displays the Pearson correlation coefficients. The correlation coefficients are coloured according to their values; blue is the positive values and red is the negative values. Correlation analysis and graphics were performed with the cor R package.

To examine the co‐variation between P‐enzymes we compared their corresponding KR‐CAP analyses results. We first limited the analysis to the P‐enzymes genes present in at least 50 samples (all but Nsap‐B and Cphy coding genes). All first KR‐CAP axes showed a highly significant positive correlation between them (Figure 3 and Table S7). The second axes of this analysis showed a different behaviour, *phoA* KR‐CAP2 showed no correlation with any other axis, *phoD* and *phoX* KR‐CAP2 displayed a similar trend between them and with a rather idiosyncratic relationship with the rest of the genes. Finally, Nsap‐A (*phoC*), Nsap‐C (*olpA*) and BPP (*phyL* and *phyS*) coding genes displayed a very similar response (Figure 3 and Table S7). No specific trend was observed according to the predicted cellular localization of the proteins.

Nsap‐B and Cphy coding genes were present in fewer samples, so we compared them to the other nonspecific acid phosphatase and phytase, respectively. For Nsap‐B, the KR‐CAP1 axis was significantly correlated to the KR‐CAP2 axes from the other two Nsap (Figure S8 and Table S7). In addition, the CPhy KR‐CAP2 axis was correlated to the BPP KR‐CAP1 axis (Figure S8 and Table S7).

Finally, when comparing each P‐enzyme CAP analysis with the subsystem level CAP analysis we observed that only *phoD* (CAP1‐PhoD vs. CAP2‐SS = 0.44), BPP (*phyL* and *phyS*) (CAP1‐BPP vs. CAP1‐SS = −0.41, CAP2‐BPP vs. CAP2‐SS = 0.55) and Cphy (*phyA*) (CAP1‐Cphy vs. CAP1‐SS = −0.59), displayed significant correlations between the axes (Table S8).

### Edge‐PCA and taxonomic identification of differentially observed P‐enzymes coding genes

Edge‐PCA analysis was applied to examine the variation in phylogenetic diversity of P‐enzyme coding genes among the soil metagenomes; a summary of the results is shown in Table S9. It is important to note that the first and second edgePCA components were highly correlated with the first and second KR‐CAP axes (except for the low abundance genes encoding *phoA* and Nsap‐B *aphA*), this enables us to connect the environmental variables to specific lineages of each gene.

**FIGURE 4:**
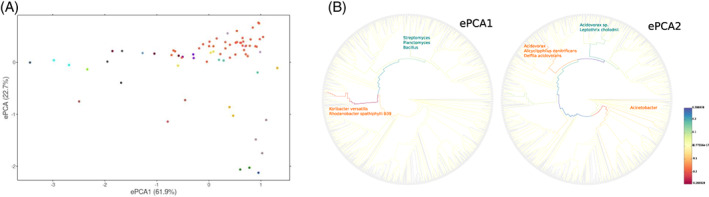
(A) Graphic representation of the first two axes of the edge‐PCA for *phoD* using samples as observations. Each point represents samples from the project mpg1992 (blue); mpg3520 (green); mpg5588 (dark red); mpg7792 (grey); mpg8624 (mustard); mgp9904 (violet); mgp10450 (dark blue); mgp10523 (stone blue); mgp10541 (turquoise); mgp10956 (yellow); mgp13011 (lilac); mgp13520 (jade); mpg13948(orange); mpg20922 (brown); mgp89409 (brick‐red); mgp91922 (light green); mgp93346 (light blue). (B) The phylogeny distribution of *phoD* hits along the first and second axis of the analysis (proteins with positive coefficients are marked in blue and proteins with negative coefficients are marked in orange). The edge‐PCA was performed using gappa software and tree and domain composition diagrams were drawn using Archaeopteryx (https://sites.google.com/site/cmzmasek/home/software/forester).

In the *phoD* analysis, the first edge‐PCA axis separated samples by soil type, pH and SOC content. The differences showed that the gene variants of the species *Koribcater versatilis* (class *Acidobacteriia*) and *Rhodanobacter spathiphylli* (class *Gammaproteobacteria*) (Figure 4B) were more abundant in soils classified as Ferrasols, Cambisols, Molisols/Phaeozem and Vertisol/Phaeozem with low pH and relatively high SOC content (left quadrant of Figure 4A). On the other hand, variants associated with *Actinomyces*, *Bacillus* and *Planctomyces* (Figure 4B) were more abundant in Kastanozem, Chernozem, Luvisol and Fluvisols soils with higher pH (ranged to 7.5) and lower SOC content (right quadrant of Figure 4A). The second axis was associated with *phoD* coding genes harboured by *Burkholderiales* and *Acinetobacter* with higher abundance in soils with neutral pH and low clay content (Tables S1 and S9 and Figure 4A).

The alkaline phosphatases *phoX* and *phoA* showed a narrower phylogenetic distribution and *Alphaproteobacteria* (*Rosevivax* and *Agrobacterium* among others) genes were predominant in soils with high SOC values and relatively high *T*_max_ (23°C) (Table S1 and Table S9). The *Burkholderiales* variants were observed in soil samples with near‐neutral pH and average SOC and CEC values (Figure S9). The genes *phoA* of *Pantoea* and *Providencia* together with *Acinetobacter* and *Actinobacter* genera were associated with varying abundance between samples (Figure S9). Again, *Acinetobacter* was differential and more abundant in soils with *circum*‐neutral pH and average SOC and CEC values (Figure S9).

We identified the acid phosphatases Nsap‐A coding genes harboured by *Pedosphaera*, *Dyella jiangningensis* and *Dyella japonica* as the differentials and the most abundant among soils with average SOC and CEC values and sandy texture (Figure S9). On the other hand, *Sphingomonas* sp., *Phenylobacterium* sp., *Rhodanobacter* sp. and *Caulobacter* species variants were more abundant in soils with high clay content (Figure S9).

The Nsap‐C coding genes harbouring by *Stenotrophomonas* (*Gammaproteobacteria*) and *Pedobacter* genera were differential and the most abundant in soils with low values of soil moisture and actual evapotranspiration (aet). *Enterobacter* Nsap‐B coding genes variants predominated in soils classified as Ferralsols, Andosols and Luvisol with acidic pH (range to 5), and high aet and precipitation (ppt) values. Metagenomes from Fluvisol, with a pH = 7, were associated with P‐enzymes gene encoding variants of *Photobacter* and *Marinomonas* and were strikingly different from the rest (Figure S9).

We only found the BPP phytase coding genes in soil samples with pH values above 6.6. BPP coding gene variants of the *Acinetobacter*, *Pseudomonas*, *Methylophaga*, *Pseudoalteromonas* and *Alteromonadales* (*Gammaproteobacteria*), and *Shewanella* and *Hylemonella* (*Betaproteobacteria*), dominate in clay soils with high CEC values. BPP genes harboured by *Bacillus* species were most abundant in sandy soils with low nutrient content.

CPhy coding genes from *Beta* and *Deltaproteobacteria*, *Clostridia* and several genera of *Negativicutes* classes varied across the samples but there was no clear signal to reveal associations with environmental variables.

## DISCUSSION

Soil ecosystems include complex interrelations among different factors including soil types, plant communities, microbial communities (bacteria, fungi, archaea, viruses and protozoa), macro and micro fauna, environmental variables, etc. (Islam et al., 2020). The present work focused on the Bacterial fraction of the soil microbial community from grassland biomes, in particular the abundance and phylogenetic diversity of P‐enzyme coding genes from the grassland biomes, using a metagenomic approach. The analysed samples represent different environmental conditions defined by the physical and chemical soil properties, and climate variables (Amundson, 2013; Islam et al., 2020). We included publicly available data from MG‐RAST and other sources for each project/sample. Other interesting data, such as the composition of the plant community or short‐term/long‐term experiments, was not included, which can constitute an interesting input for the analyses and discussion of our study.

Microbial P enzymes, such as phosphatases (Nannipieri et al., 2011; Rodriguez et al., 2006) and phytases (Tan et al., 2013; Yao et al., 2012), play a crucial role in the phosphorus cycle by participating in the release of Pi from organophosphorus compounds, the last step of the P cycle (Zeng et al., 2022). One valuable result of this study is that it confirms and expands the idea of the large variability in abundance and diversity of P‐enzymes coding genes within grassland ecosystems across the planet.

Our analyses showed that the alkaline phosphatases were the most abundant P‐enzymes genes in the whole dataset, being the *phoD* gene the most abundant and also with the widest phylogenetic distribution, regardless of the soil properties. This result is in accordance with reports by previous, but more restricted, metagenomic studies where this gene was the most frequently alkaline phosphatase found in different soils (Bergkemper et al., 2016; Park et al., 2022; Tan et al., 2013). The *phoA* was less abundant, and the difference with *phoD* or *phoX* can result from the differences in substrate specificity and co‐factor requirements between them. Bacterial cells may possess either *phoX* or *phoA* or both. They are presumed to have similar roles in facilitating access to a diverse array of phosphoester compounds and are more active against organic phosphates and nucleotides. Nonetheless, they may function at varying levels of substrate concentrations (Sebastian & Ammerman, 2011). A study about PhoA activity in marine ecosystems showed that this enzyme has an activity for mono‐, di‐ and triesterase activity (Srivastava et al., 2021). PhoX is essential for utilizing monophosphate esters at low substrate concentrations in *Rhizobium pomeroyi* (Sebastian & Ammerman, 2011). The substrate specificity of PhoD is unknown. Some work has reported phosphodiesterase activity against cell wall teichoic acids and phospholipids (Bergkemper et al., 2016; Rodriguez et al., 2014). However, the contribution to the Pho‐regulated phosphatase activity of *Pseudomonas fluorescens* does not seem to be significant (Monds et al., 2006). A new alkaline phosphatase, PafA, has recently been described in plant‐associated Bacteroidetes (Lidbury et al., 2021). Unlike PhoD, PhoX and PhoA, this enzyme exhibits constitutive phosphatase activity and is fully functional in the presence of high phosphate concentrations with high monophosphatase activity. PafA plays a critical role in global biogeochemical cycles and has potential applications in sustainable agriculture (Lidbury et al., 2021).

On the other extreme, genes encoding Nspa‐B and Cphy were scarce in the whole dataset. These genes tend to show weaker associations with the environmental variables and other P‐enzyme coding genes. This could be due to the low numbers in which these genes appear or to genuine biological reasons.

### Environmental variables and P‐enzyme coding genes abundance and diversity

We showed that several environmental variables are related to the diversity and abundance of P‐enzyme coding genes. *T*_max_, pH, SOC and soil moisture are associated with alkaline phosphatase gene abundance. The *phoD* and *phoX* genes showed a high correlation with SOC and clay. Several recent studies report the effect of SOC, N and organic P content on the abundance and diversity of both enzymes and the corresponding bacteria (Li et al., 2021; Ragot et al., 2017; Wei et al., 2021). A local‐scale study of three land uses with differential SOC (fallow, arable, grassland) demonstrated there was a positive correlation between alkaline phosphatases gene abundance and soil organic matter contents (Neal et al., 2017). In addition, the predicted extracellular location of both enzymes (Neal et al., 2017) may explain the importance of clay content in relation to its stabilization role, immobilization and maintenance of the enzymatic activity (Margalef et al., 2017). The *phoD* genes are widely distributed among different classes of Bacteria, in this study we found that variants associated with *Koribacter* (*Acidobacteria* class) and *Rhodanobacter* genus (*Gammaproteobacteria* class) were more abundant in soils with relatively high SOC values and low pH. These variants have been identified as a dominant phylotype in arable silty clay loam soil Chromic Luvisol in the United Kingdom (Neal et al., 2017). The second one also has been identified as a dominant phylotype in the rhizospheres of maize and sorghum in a Brazilian Distroferric Red Latosol (Neal et al., 2021). Both bacterial species represent classes that possess a comprehensive set of genes that allow them to use a wide variety of substrates, responding efficiently to environmental changes and conferring their ability to adapt to various ecological niches (Kalam et al., 2020; Kurm et al., 2017). Variants associated with *Bacillus*, *Actinomyces* and *Planctomyces* were prevalent in soils with lower SOC and neutral pH. The last two species have been found dominant in soils with low nutrient content, even the *Planctomyces* showed a negative correlation with this variable (Garaycochea et al., 2020; Hermans et al., 2017; Lewin et al., 2017). Nevertheless, the main driver that explains the difference in species abundances appears to be pH, since all reported species are heterotrophs (Kielak et al., 2016; Saxena et al., 2020). The *phoX* gene represented by the *Burkholderia* genus was preferred in soils with low and medium content of SOC and neutral pH. Bacteria from this genus present a wide repertoire of metabolic pathways making them more competitive in nutrient‐restrictive environments, since they are capable to degrade recalcitrant compounds, and unlike most Bacteria, *Burkholderia* species are more competitive in low and moderate pH conditions (Morya et al., 2020; Stopnisek et al., 2014).

Regarding acid phosphatases, the Nsap‐A coding genes were found in *Dyella* and *Rhodanobacter* genera. These species use different carbon sources and have been reported to be dominant in acid and neutral soils (Dahal & Kim, 2017; Weon et al., 2009). On the other hand, the Nsap‐C coding gene was identified in *Alpha* and *Gammaproteobacteria*, *Flavobacteria* and *Sphingobacteria* classes, consistent with previous evidence (Gaiero et al., 2020; Neal et al., 2017). The proportion of both non‐specific acid phosphatases found in the grassland set studied here was similar to that reported for UK grassland soils (Neal et al., 2017). The predominance of acid phosphatases in grassland could be influenced by the interaction between microorganisms and plant communities, as both are capable of producing these enzymes (Mhlongo et al., 2018). The observed proportion of Nsap‐B is similar to that reported by Udaondo et al. (2020), who not only found that this enzyme was less abundant in different niches but also that it was restricted to a limited number of microbial families, some of which were pathogens.

In the cases of phytases, BPP coding genes showed an abundance and phylogenetic distribution in accordance with what has been reported. The BPP coding genes are widespread and are distributed among various species of soil bacteria (Huang et al., 2009; Jorquera et al., 2008; Kumar et al., 2017; Lim et al., 2007). However, some studies have observed that the presence of BPP coding genes is rare in *Betaproteobacteria* (Cotta et al., 2016), we found that the BPP coding genes variants were mainly from *Bacillales* and *Beta* and *Gammaproteobacteria*. The BPP coding genes in this study were found restricted to soil with pH above 6.6, which is in accordance with what was reported, particularly in several strains from the *Bacillus* genus, where the BPPs enzymes are optimally active at pH 6.0–7.5 (Cheng & Lim, 2006; Farhat et al., 2008; Huang et al., 2009; Kerovuo et al., 1998; Kumar et al., 2017). On the other hand, the Cphy coding gene was the least abundant enzyme in the grasslands metagenomes, contrary to those found by Neal et al., 2017 where CPhy tended to have a similar abundance that BPP in the studied grasslands from the UK.

The pH appears as an important factor associated with both acid and alkaline phosphatases, as well as phytases, abundance and diversity. Even though, our results show a global trend of an increase in the genes encoding these enzymes (PhoD, PhoX, Nspa‐C and BPP) with pH, all enzymes are relatively abundant in the pH range covered in this study, rendering it difficult to test a direct association between the enzyme classification (as acid or alkaline) and the soil pH.

It is important to bear in mind that the taxonomic associations of each gene sequence are dependent on the database, is clear that including different sequences of more taxa might result in the discovery of new variants and/or better assignments of the sequences. Nevertheless, many of the results here obtained will still hold being enriched with the new putative ones.

### Co‐variation of P‐enzyme coding genes

We have shown a strong relationship in the abundance and diversity patterns between the different P‐enzymes herein studied. Indeed, KR‐CAP analyses show strong correlations between them, somehow weaker in the less abundant genes. These results uncover a somehow intuitive result. We should bear in mind that we are counting the aggregate of each gene in a whole community, thus, variation in abundance and diversity of a given gene is the product of a change at the community level. So the process of selection in the assemblage of each community is a balance between how each organism crafted its genome and the interaction between them and the environment. The high correlation between KR‐CAP analyses, which involve abundance, diversity and environmental variables, suggests a tight relationship between the P‐enzyme genes. This implies that for each environmental condition, the way each P‐enzyme gene contributes to phosphorus cycling and metabolism is connected to the rest of them (in both abundance and diversity). The different taxa that appear associated with each P‐enzyme coding gene in the edgePCA analysis (Table S9) are indicative that different organisms are contributing to the P‐enzyme gene pool.

Another interesting result was the association of P‐enzymes with the general functional profiles (Table S8). Here, the results are somehow at odds with the previous one. The first axes of PhoD, PhoX, PhoA and BPP coding genes were strongly correlated with the CAP2 of the functional profiles. Nevertheless, Cphy and Nsap‐X genes, showed no correlation, suggesting that there could be some variability in this respect.

One important question is to understand if the P‐enzymes are driven particularly by the change of certain organisms that are carrying them or, in turn, they are following the general major changes in the community structure. One possible hint in this direction is given by the previous comparison, indicating that these P‐enzymes genes may be accompanying the general change in the functional structure of the metagenome, whilst there is room for a more idiosyncratic manner. Nevertheless, more studies should be carried out to gain deeper insight into this interesting and complex question.

### Concluding remarks

The environmental variables explained a relatively low proportion of the variability in bacterial functional profiles. The use of information from samples from very distant sites determines only the effect on the diversity of the variables with greater differences among the sites. However, *T*_max_, soil pH and evapotranspiration were related to the abundance and diversity of almost the eight key enzymes involved in P organic cycling. Likewise, it was possible to identify the effect of other variables with a more localized effect, such as soil texture and soil organic content, as important determinants of microbial community structure and functions. The complexity of the studied system requires a combination of approaches and the generation of local data that allow the understanding of factors affecting the presence of bacteria carrying P‐enzymes genes as well as their functionality and to integrate these results into a broader scale to detect global patterns of diversity that could potentially lead to better understanding and management of soil P cycling.

## AUTHOR CONTRIBUTIONS

**Silvia Garaycochea:** Conceptualization (equal); data curation (equal); formal analysis (equal); funding acquisition (supporting); investigation (equal); methodology (equal); resources (equal); writing – original draft (equal); writing – review and editing (equal). **Nora Adriana Altier:** Conceptualization (supporting); funding acquisition (lead); project administration (lead); writing – original draft (supporting). **Carolina Leoni:** Formal analysis (supporting); methodology (supporting); writing – original draft (supporting). **Andrew L. Neal:** Conceptualization (supporting); methodology (supporting); writing – original draft (supporting). **Héctor Romero:** Conceptualization (equal); data curation (equal); formal analysis (equal), methodology (supporting), writing – original draft (equal), writing – review and editing (equal).

## CONFLICT OF INTEREST

The authors declare no conflict of interest.

## Supporting information


**FIGURE S1.** Geographical distribution of the samples included in this work. A total of 376 grasslands samples from 17 project around the word selected through the TerrestrialMetagenomeDB (https://webapp.ufz.de/tmdb/). This map was created this R package ggmap (Kahle and Wickham, 2013).

**FIGURE S2.** CAP based on Mahalanobis distance of subsystems MG‐Rast annotations for grassland subset (n = 74) . PERMANOVA analysis with 999 permutations was performed to determine the significance between the sites/MG‐Rast project. For each MG‐Rast project were included three samples with the same geo‐reference. The variable's vector length represents the correlation between each variable and the axes. On the first plot the samples are colored by project and the second one by soil type.

**FIGURE S3.** Phylogenetic placements tree to the metagenomes predicted proteins showing homology with enzymes PhoA, Cphy, Nsap‐B and Nsap‐C reference database The size of the circle representing placements proportional to the abundance.

**FIGURE S4.** CAP based on Mahalanobis distance of enzyme abundance on grassland subset (n = 74). PERMANOVA analysis with 999 permutations was performed to determine the significance between the sites/MG‐Rast project. The variable's vector length represents the correlation between each variable and the axes. On the first plot the samples are colored by project and the second one by soil type.

**FIGURE S5.** CAP based on Kantorovich‐Rubinstein distance for alkaline phosphatases. PERMANOVA analysis with 999 permutations was performed to determine the significance between the sites/project. The variable's vector length represents the correlation between each variable and the axes. To PhoA and PhoX showed two plots, in the first one the samples are marked with color by project and in the second colored by soil type. The PhoD is colored by soil type

**FIGURE S6.** CAP based on Kantorovich‐Rubinstein distance for non‐specific acid phophatases (Nsap). PERMANOVA analysis with 999 permutations was performed to determine the significance between the sites/project. The variable's vector length represents the correlation between each variable and the axes. For each Nsap were showed two plots, in the first one the samples are colored by project and in the second colored by soil type.

**FIGURE S7.** CAP based on Kantorovich‐Rubinstein distance for Phytases. PERMANOVA analysis with 999 permutations was performed to determine the significance between the sites/project. The variable's vector length represents the correlation between each variable and the axes. For each phytase were showed two plots, in the first one the samples are colored by project and in the second colored by soil type.

**FIGURE S8.** Correlation matrix of KR‐CAP axes. The correlograms displays the Pearson correlation coefficients among the less abundant P‐enzymes: acid phosphatases: KR‐CAP Nsap‐A axes and KR‐CAP Nsap‐B axes; KR‐CAP Nsap‐C axes and KR‐CAP Nsap‐B axes and phytase KR‐CAP BPP axes and KR‐CAP CPhy. The correlation coefficients are colored according to their values; being blue the positives values and red the negative values. The correlations are significant at *0.01; **0.05; ***0.001. Correlation analysis and graphics were performed with cor R package.

**FIGURE S9.** Graphic representation of the first two axes of the edge‐PCA for each enzyme using samples as observations. Each point represents samples from the project mpg1992 (blue); mpg3520 (green); mpg5588 (dark red); mpg7792 (gray); mpg8624 (mustard); mgp9904 (violet); mgp10450 (dark blue); mgp10523 (stone blue); mgp10541 (turquoise); mgp10956 (yellow); mgp13011 (lilac); mgp13520 (jade); mpg13948(orange); mpg20922 (brown); mgp89409 (brick‐red); mgp91922 (light green); mgp93346 (light blue). (b) The phylogeny distribution of each enzyme hits along the first and second axis of the analysis (protein with positive coefficients are marked in blue and proteins with negative coefficients are marked in orange).

**TABLE S1.** Metadata associated to set 1 samples

**TABLE S2.** Functional annotation based on subsystems MG‐Rast

**TABLE S3.** Metadata associated to grassland set samples

**TABLE S4.** Functional annotation based on subsystems MG‐Rast

**TABLE S5.** Enzyme relative abundance

**TABLE S6.** Correlation analysis between enzymes abundance and environmetal variables

**TABLE S7.** Pearson correlation analysis among CAP axes 1 and 2

**TABLE S8.** Pearson correlation analysis among CAP axes 1 and 2 most abundant enzymes coding genes and Subsytems annotation

**TABLE S9.** List of bacterial classes differential for each metagenomes from edge PCA analysis

## Data Availability

All data used in this work are publicly available. Additional data, additional results and scripts are available at https://github.com/eletor-uy/Grasslands.
