# Supplementary figures and images for: Abundance and phylogenetic distribution of eight key enzymes of the phosphorus biogeochemical cycle in grassland soils
Source: Environ Microbiol Rep. 2023 May 10;15(5):352–69. doi: 10.1111/1758-2229.13159 (PMC10472533; doi:10.1111/1758-2229.13159)

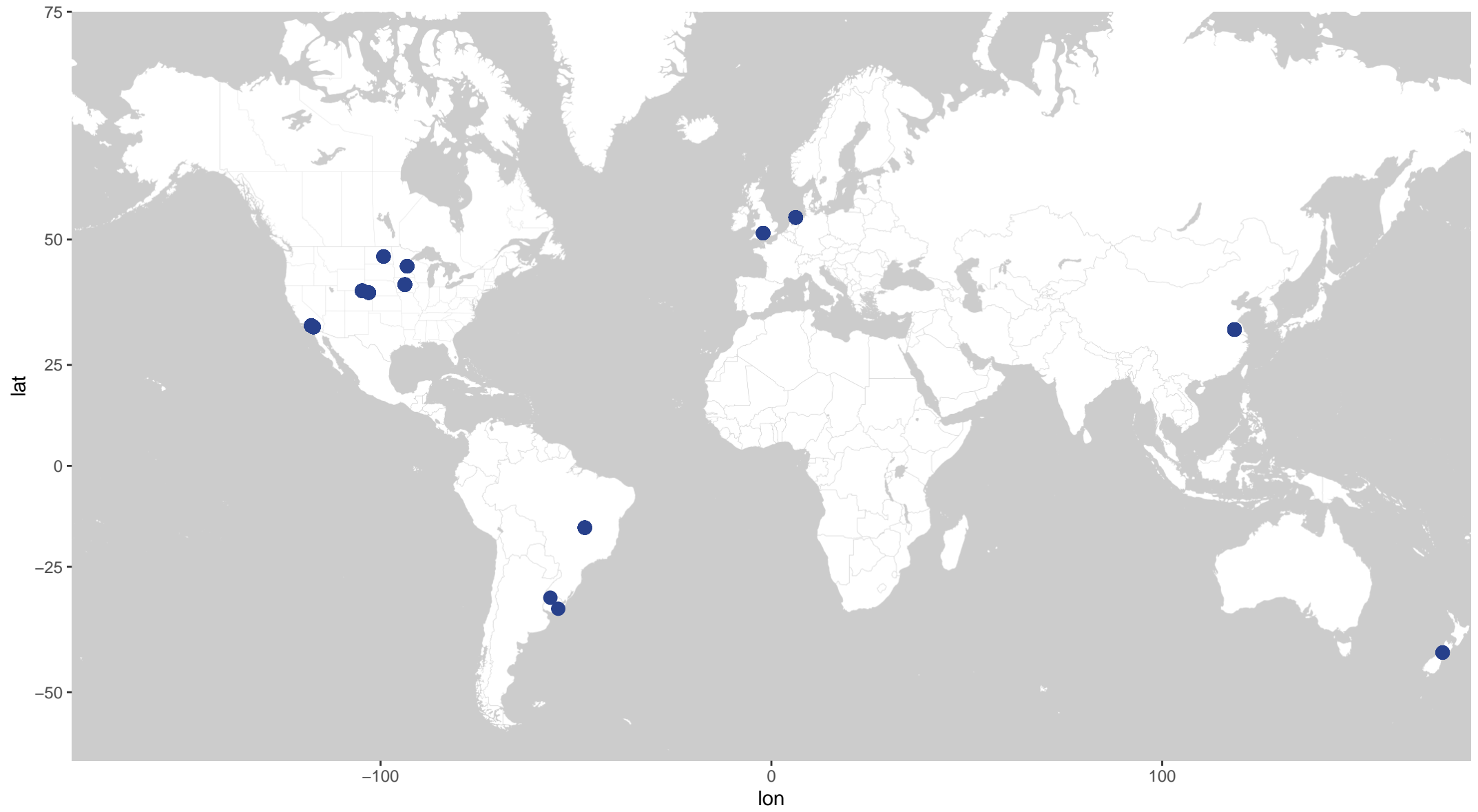

Supplement: Supplementary file 1 — FIGURE S1. Geographical distribution of the samples included in this work. A total of 376 grasslands samples from 17 project around the word selected through the TerrestrialMetagenomeDB (https://webapp.ufz.de/tmdb/). This map was created this R package ggmap (Kahle and Wickham, 2013). [file EMI4-15-352-s016.pdf]

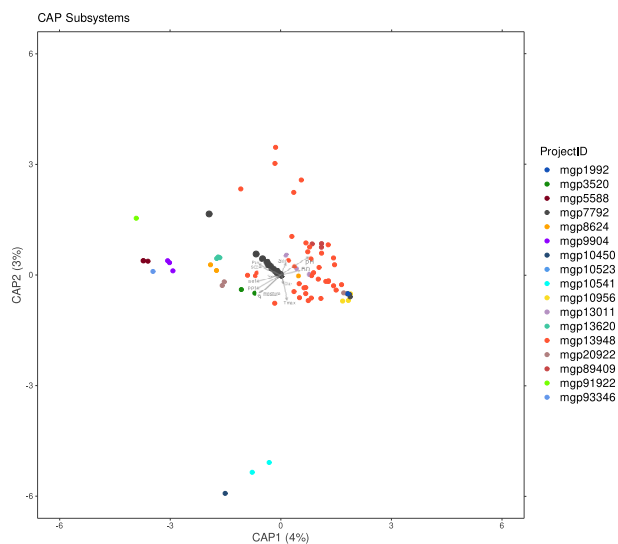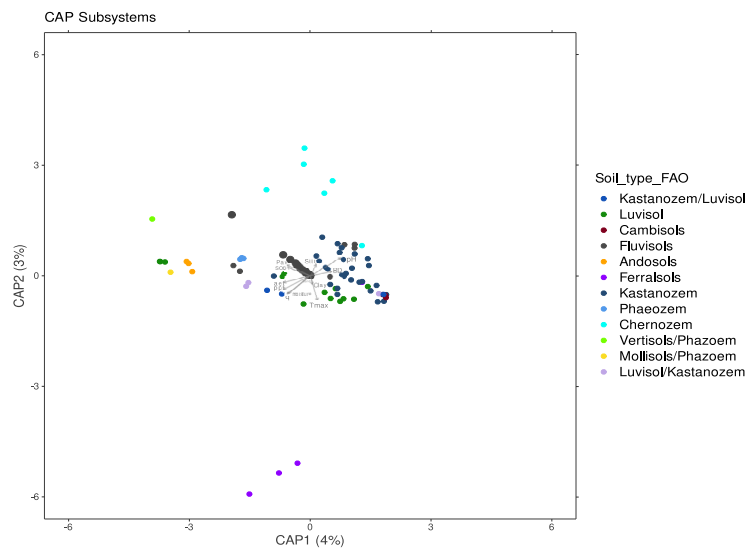

Supplement: Supplementary file 2 — FIGURE S2. CAP based on Mahalanobis distance of subsystems MG‐Rast annotations for grassland subset (n = 74) . PERMANOVA analysis with 999 permutations was performed to determine the significance between the sites/MG‐Rast project. For each MG‐Rast project were included three samples with the same geo‐reference. The variable's vector length represents the correlation between each variable and the axes. On the first plot the samples are colored by project and the second one by soil type. [file EMI4-15-352-s012.pdf]

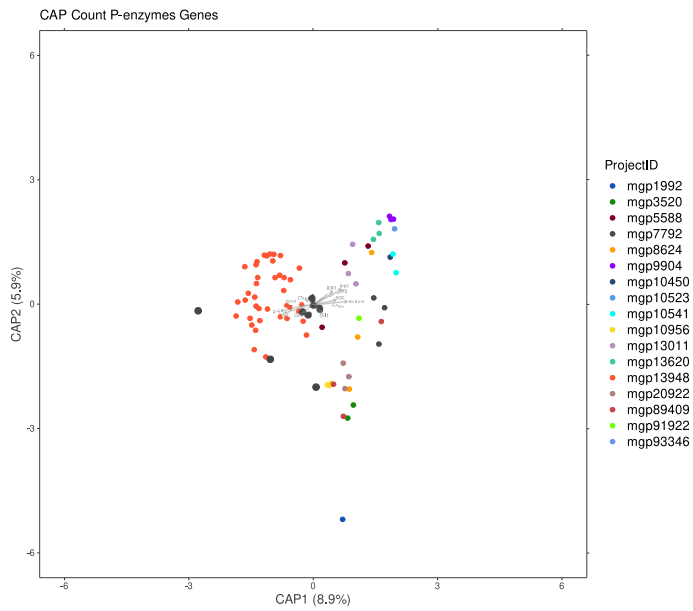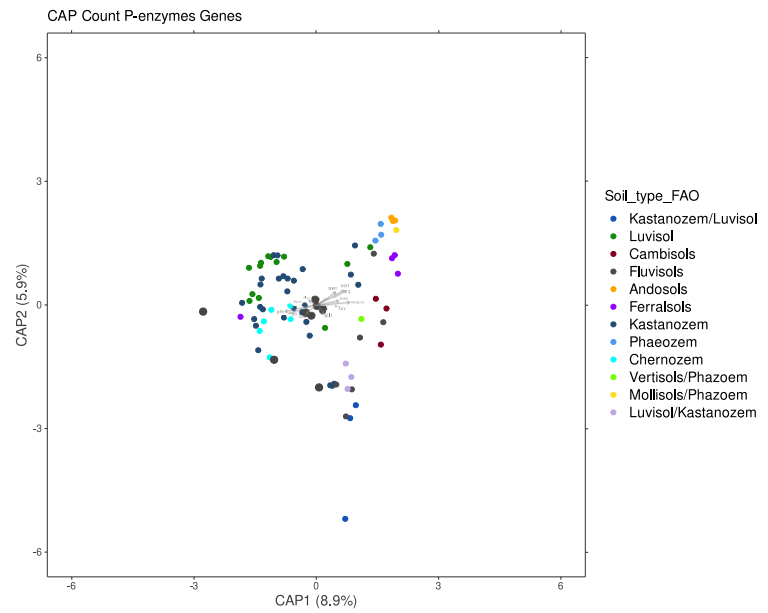

Supplement: Supplementary file 4 — FIGURE S4. CAP based on Mahalanobis distance of enzyme abundance on grassland subset (n = 74). PERMANOVA analysis with 999 permutations was performed to determine the significance between the sites/MG‐Rast project. The variable's vector length represents the correlation between each variable and the axes. On the first plot the samples are colored by project and the second one by soil type. [file EMI4-15-352-s009.pdf]

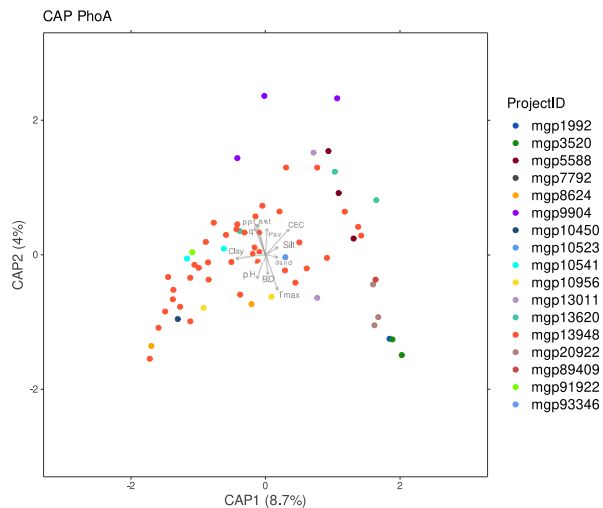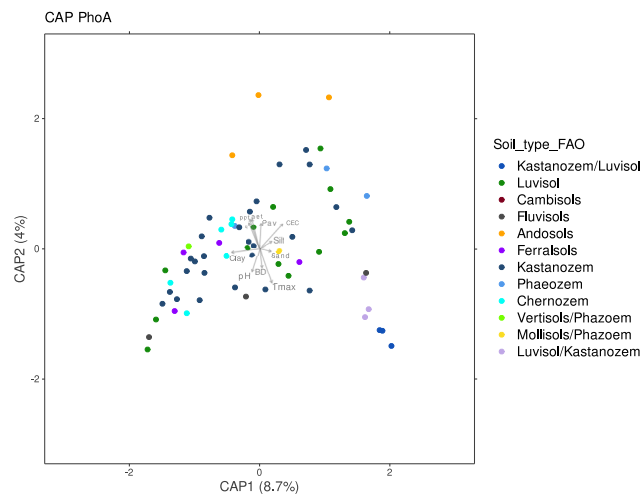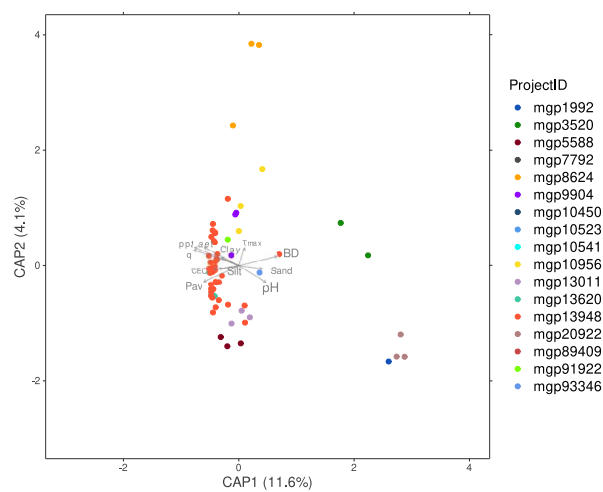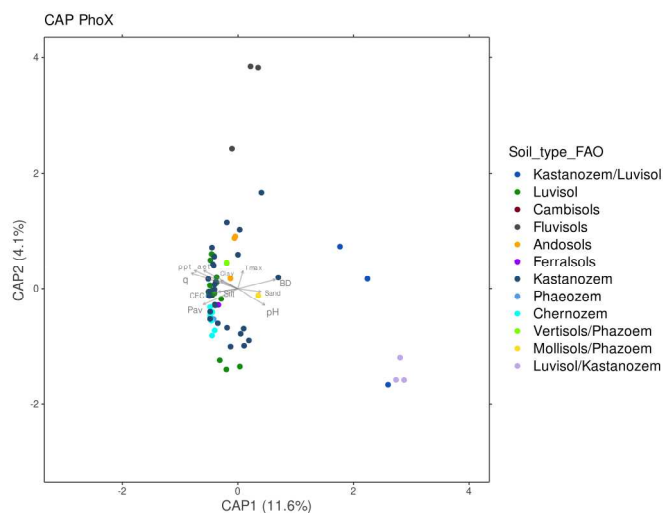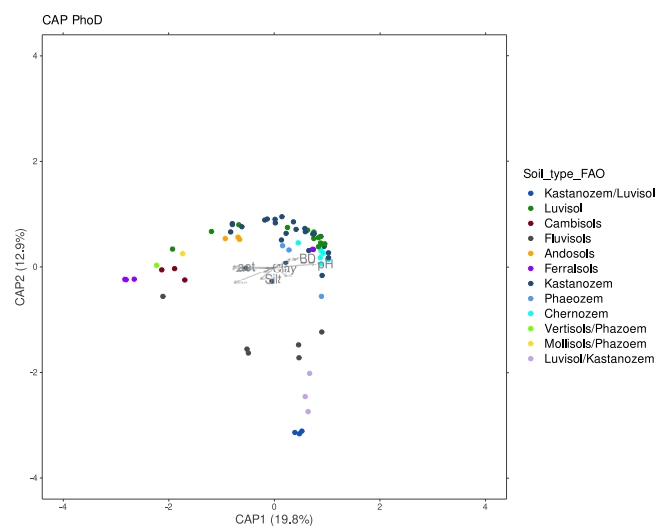

Supplement: Supplementary file 5 — FIGURE S5. CAP based on Kantorovich‐Rubinstein distance for alkaline phosphatases. PERMANOVA analysis with 999 permutations was performed to determine the significance between the sites/project. The variable's vector length represents the correlation between each variable and the axes. To PhoA and PhoX showed two plots, in the first one the samples are marked with color by project and in the second colored by soil type. The PhoD is colored by soil type [file EMI4-15-352-s001.pdf]

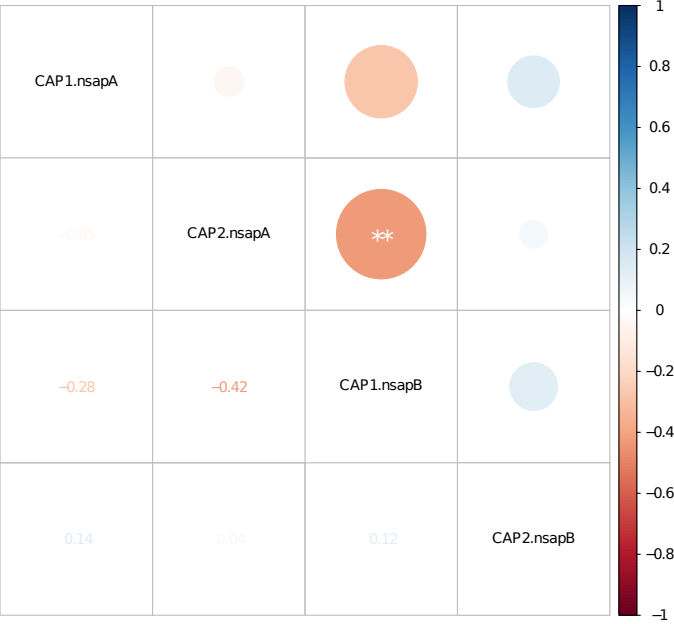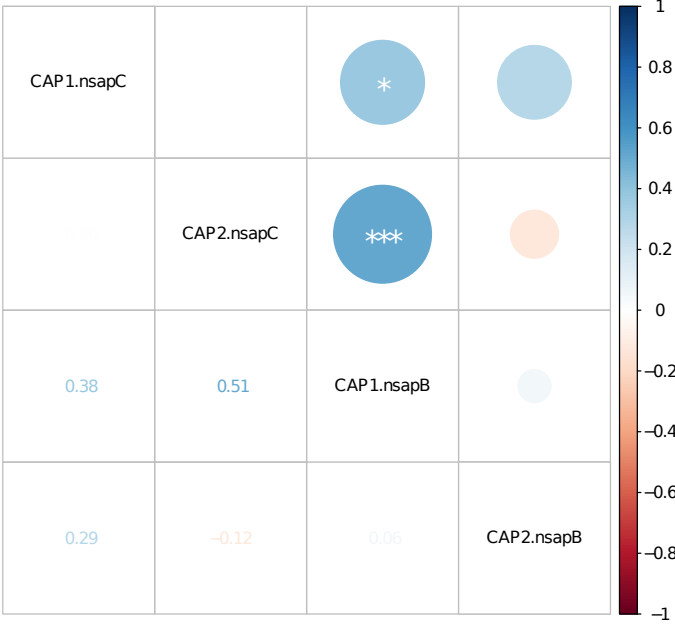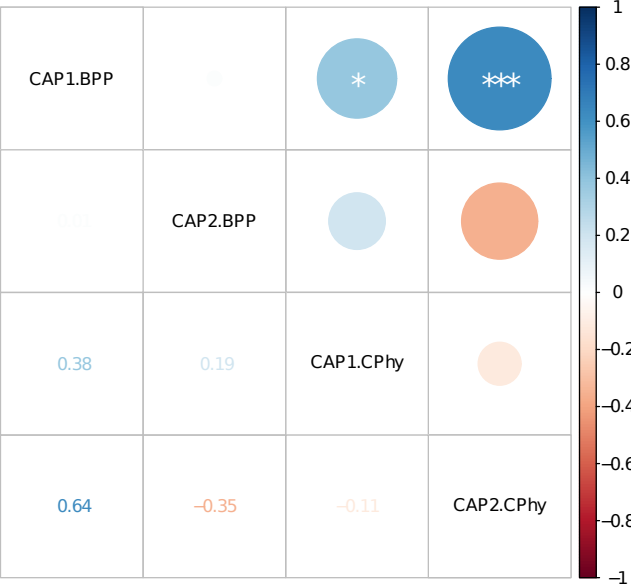

Supplement: Supplementary file 8 — FIGURE S8. Correlation matrix of KR‐CAP axes. The correlograms displays the Pearson correlation coefficients among the less abundant P‐enzymes: acid phosphatases: KR‐CAP Nsap‐A axes and KR‐CAP Nsap‐B axes; KR‐CAP Nsap‐C axes and KR‐CAP Nsap‐B axes and phytase KR‐CAP BPP axes and KR‐CAP CPhy. The correlation coefficients are colored according to their values; being blue the positives values and red the negative values. The correlations are significant at *0.01; **0.05; ***0.001. Correlation analysis and graphics were performed with cor R package. [file EMI4-15-352-s002.pdf]
