# Supplementary material for: Abundance and phylogenetic distribution of eight key enzymes of the phosphorus biogeochemical cycle in grassland soils
Source: Environ Microbiol Rep. 2023 May 10;15(5):352–69. doi: 10.1111/1758-2229.13159 (PMC10472533; doi:10.1111/1758-2229.13159)
Supplement: Supplementary file 3 — FIGURE S3. Phylogenetic placements tree to the metagenomes predicted proteins showing homology with enzymes PhoA, Cphy, Nsap‐B and Nsap‐C reference database The size of the circle representing placements proportional to the abundance. [file EMI4-15-352-s007.pdf]

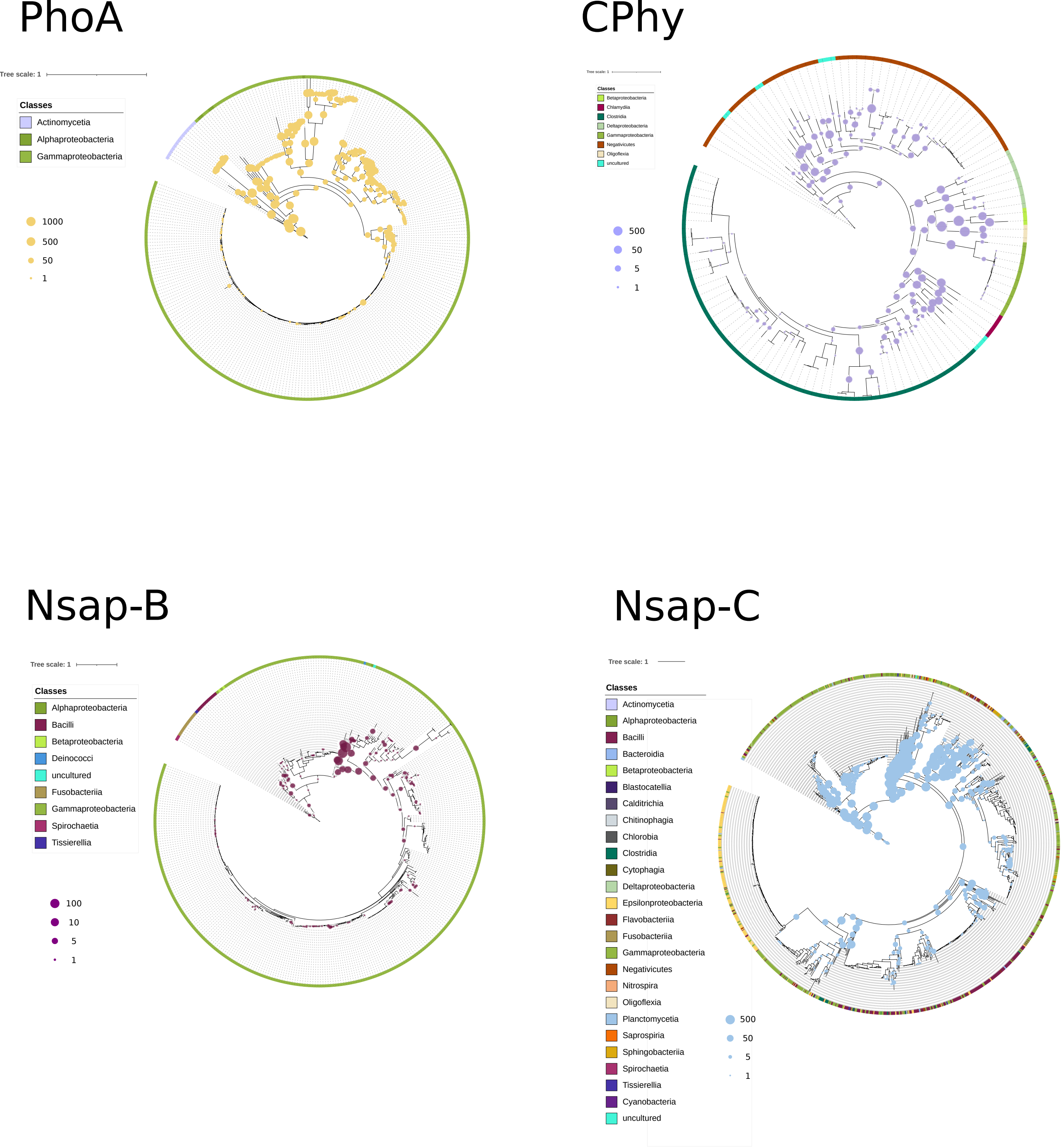
PhoA
Tree scale: 1
Classes
Actinomycetia
Alphaproteobacteria
Gammaproteobacteria
1000
500
50
1
CPhy
Tree scale: 1
Classes
Betaproteobacteria
Chlamydiia
Clostridia
Deltaproteobacteria
Gammaproteobacteria
Negativicutes
Oligoflexia
uncultured
500
50
5
1
Nsap-B
Tree scale: 1
Classes
Alphaproteobacteria
Bacilli
Betaproteobacteria
Deinococci
uncultured
Fusobacteriia
Gammaproteobacteria
Spirochaetia
Tissierellia
100
10
5
1
Nsap-C
Tree scale: 1
Classes
Actinomycetia
Alphaproteobacteria
Bacilli
Bacteroidia
Betaproteobacteria
Blastocatellia
Calditrichia
Chitinophagia
Chlorobia
Clostridia
Cytophagia
Deltaproteobacteria
Epsilonproteobacteria
Flavobacteriia
Fusobacteriia
Gammaproteobacteria
Negativicutes
Nitrospira
Oligoflexia
Planctomycetia
Saprospiria
Sphingobacteriia
Spirochaetia
Tissierellia
Cyanobacteria
uncultured
500
50
5
1
