# Supplementary material for: Abundance and phylogenetic distribution of eight key enzymes of the phosphorus biogeochemical cycle in grassland soils
Source: Environ Microbiol Rep. 2023 May 10;15(5):352–69. doi: 10.1111/1758-2229.13159 (PMC10472533; doi:10.1111/1758-2229.13159)

PhoX

ePCA1

Sphaerotilus natans
Variovorax paradoxus
Ramlibacter tataouinensis
Leptothrix cholodnii
Candidatus Accumulibacter
Variovorax

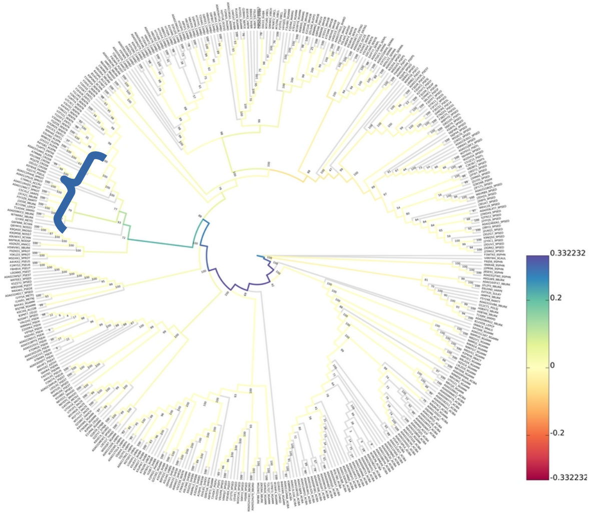

Roseivivax
halodurans

ePCA2

Variovorax sp.

Methylibium sp.

Accumulibacter phosphatis

(strain UW-1)

Leptothrix cholodnii

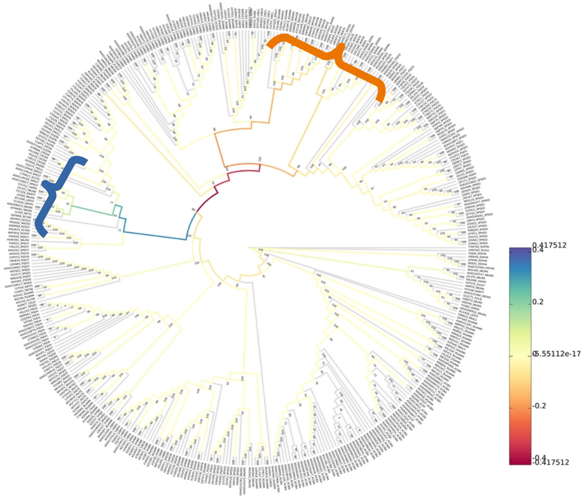

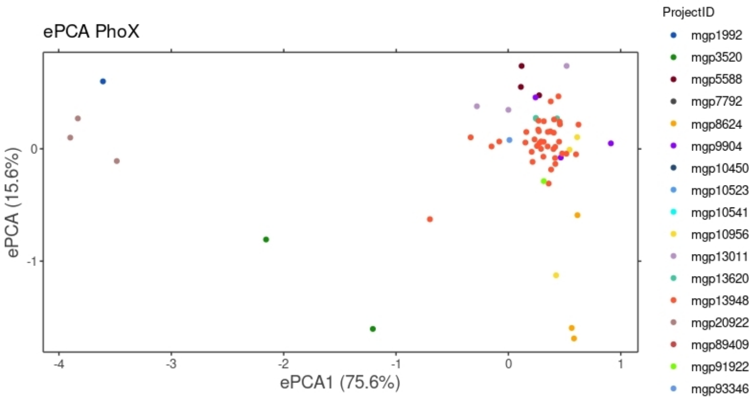

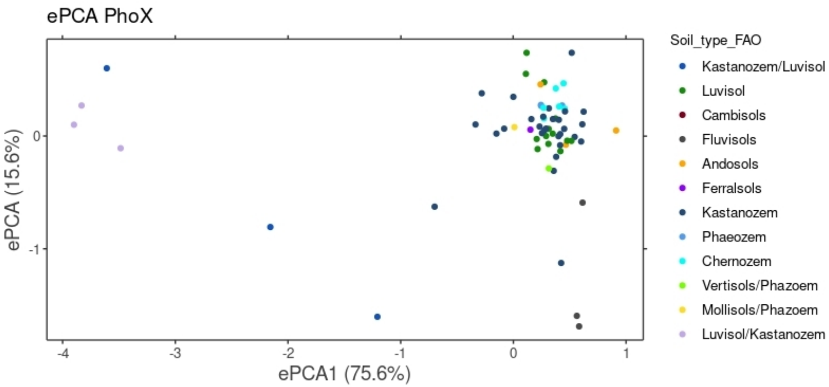

PhoA

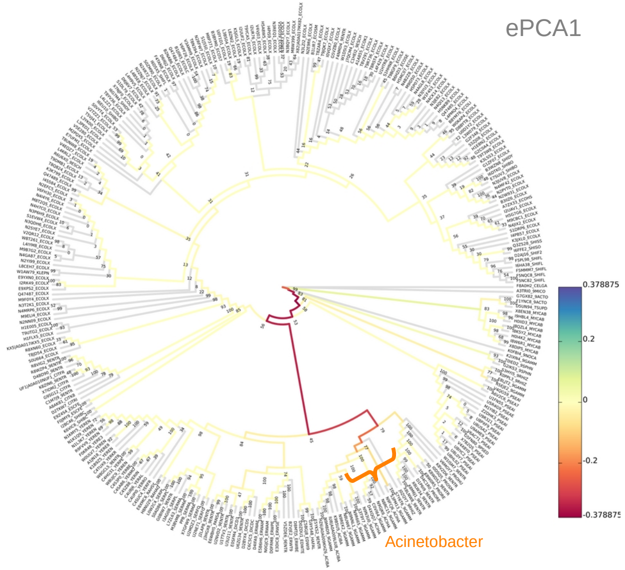
ePCA1
0.378875
0.2
0
-0.2
-0.378875
Acinetobacter

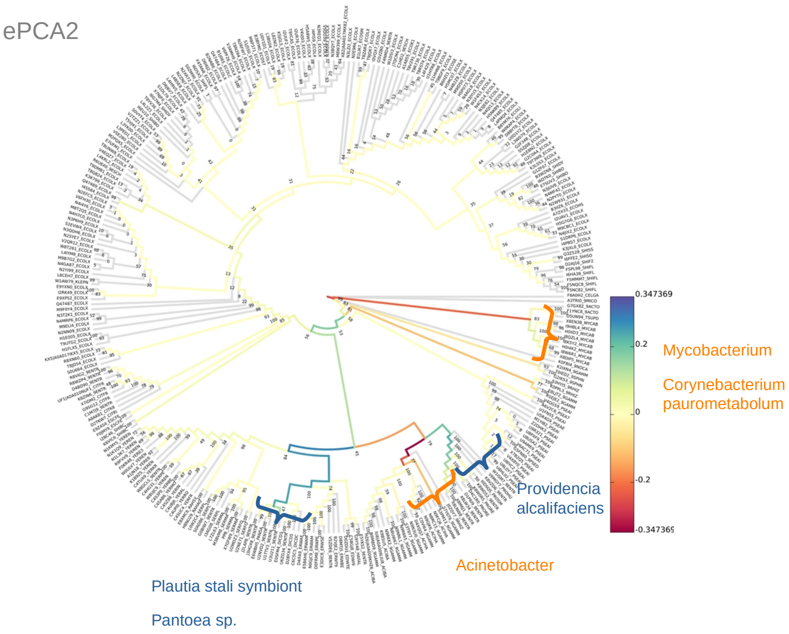
ePCA2
0.347369
0.2
0
-0.2
Mycobacterium
Corynebacterium paurometabolum
Providencia alcalifaciens
Acinetobacter
Plautia stali symbiont
Pantoea sp.

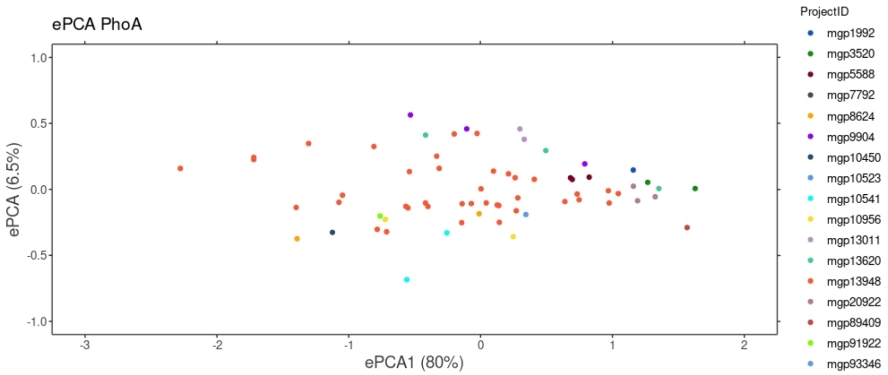
ePCA PhoA
1.0
0.5
0.0
-0.5
-1.0
-3
-2
-1
0
1
2
ePCA1 (80%)
ePCA (6.5%)
ProjectID
mgp1992
mgp3520
mgp5588
mgp7792
mgp8624
mgp9904
mgp10450
mgp10523
mgp10541
mgp10956
mgp13011
mgp13620
mgp13948
mgp20922
mgp89409
mgp91922
mgp93346

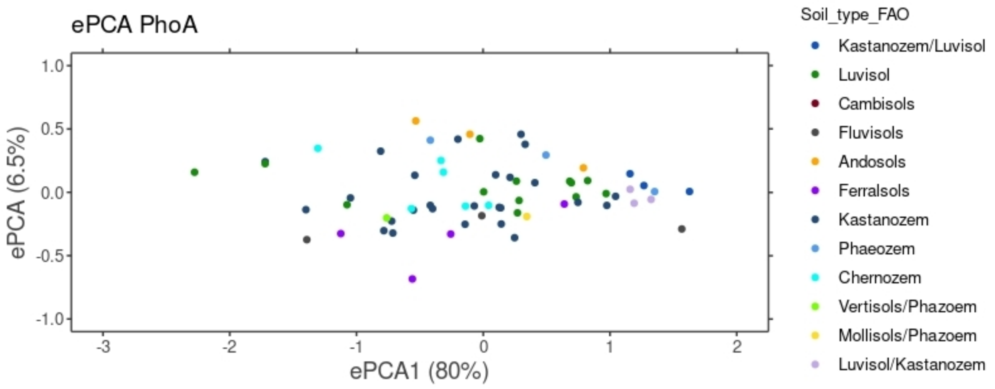
ePCA PhoA
1.0
0.5
0.0
-0.5
-1.0
-3
-2
-1
0
1
2
ePCA1 (80%)
ePCA (6.5%)
Soil_type_FAO
Kastanozem/Luvisol
Luvisol
Cambisols
Fluvisols
Andosols
Ferralsols
Kastanozem
Phaeozem
Chernozem
Vertisols/Phazoem
Mollisols/Phazoem
Luvisol/Kastanozem

nsapA

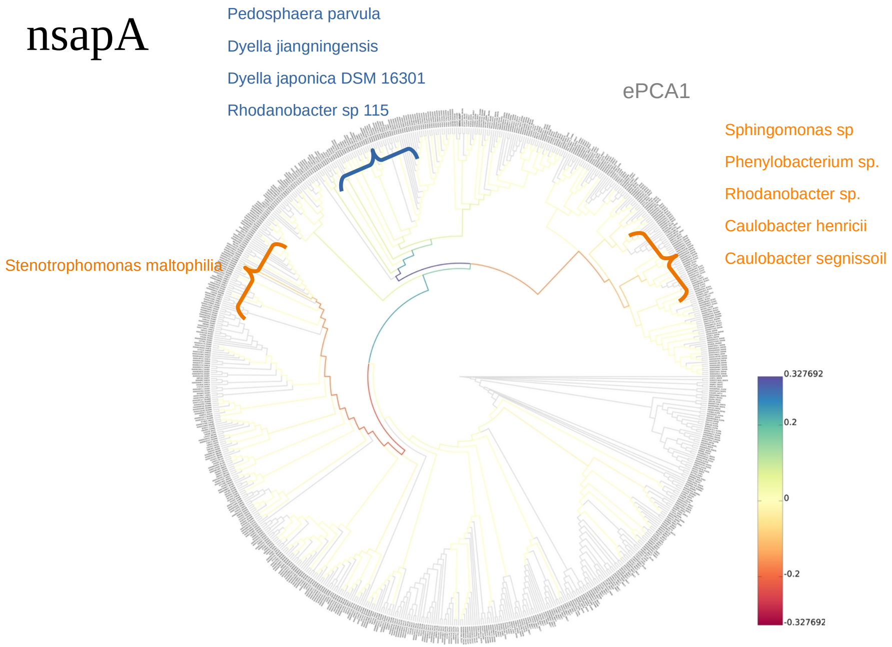
ePCA1
Pedosphaera parvula
Dyella jiangningensis
Dyella japonica DSM 16301
Rhodanobacter sp 115
Sphingomonas sp
Phenylobacterium sp.
Rhodanobacter sp.
Caulobacter henricii
Caulobacter segnissoil
Stenotrophomonas maltophilia
0.327692
0.2
0
-0.2
-0.327692

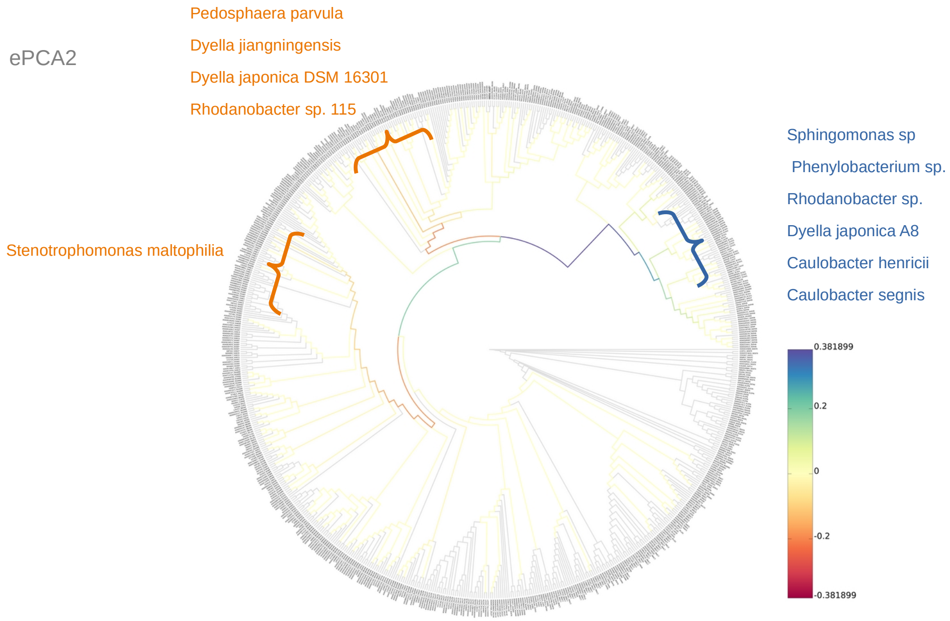
ePCA2
Pedosphaera parvula
Dyella jiangningensis
Dyella japonica DSM 16301
Rhodanobacter sp. 115
Sphingomonas sp
Phenylobacterium sp.
Rhodanobacter sp.
Dyella japonica A8
Caulobacter henricii
Caulobacter segnis
Stenotrophomonas maltophilia
0.381899
0.2
0
-0.2
-0.381899

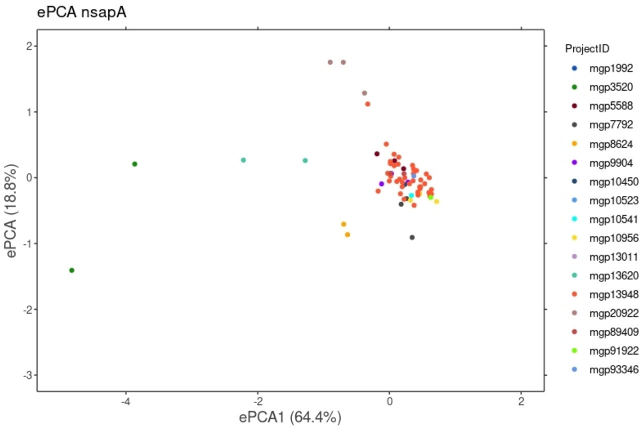
ePCA nsapA
ePCA (18.8%)
ePCA1 (64.4%)
2
1
0
-1
-2
-3
-4
-2
0
2
ProjectID
mgp1992
mgp3520
mgp5588
mgp7792
mgp8624
mgp9904
mgp10450
mgp10523
mgp10541
mgp10956
mgp13011
mgp13620
mgp13948
mgp20922
mgp89409
mgp91922
mgp93346

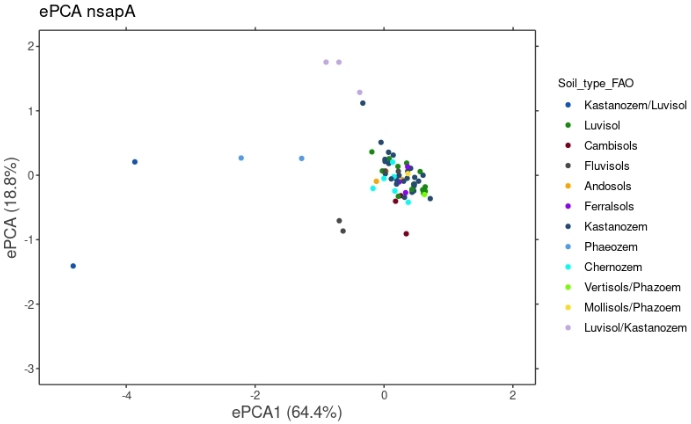
ePCA nsapA
ePCA (18.8%)
ePCA1 (64.4%)
2
1
0
-1
-2
-3
-4
-2
0
2
Soil_type_FAO
Kastanozem/Luvisol
Luvisol
Cambisols
Fluvisols
Andosols
Ferralsols
Kastanozem
Phaeozem
Chernozem
Vertisols/Phazoem
Mollisols/Phazoem
Luvisol/Kastanozem

nsapB

Enterobacter asburiae

Enterobacter cloacae

Enterobacter cloacae BWH 31

Enterobacter

Enterobacter sp

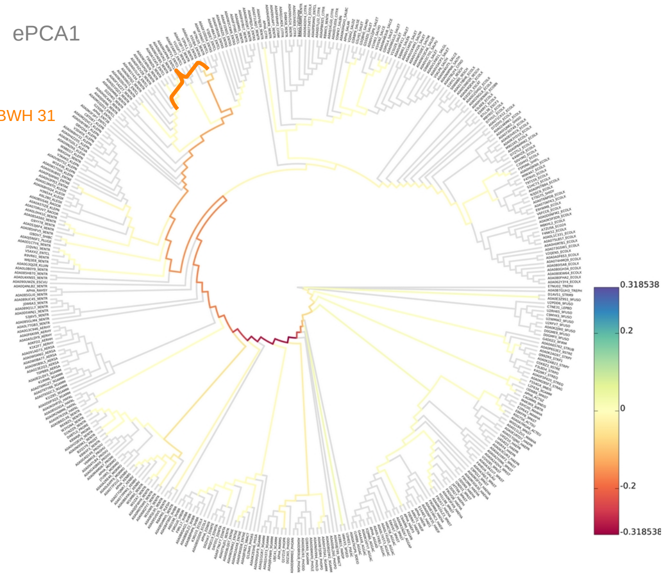

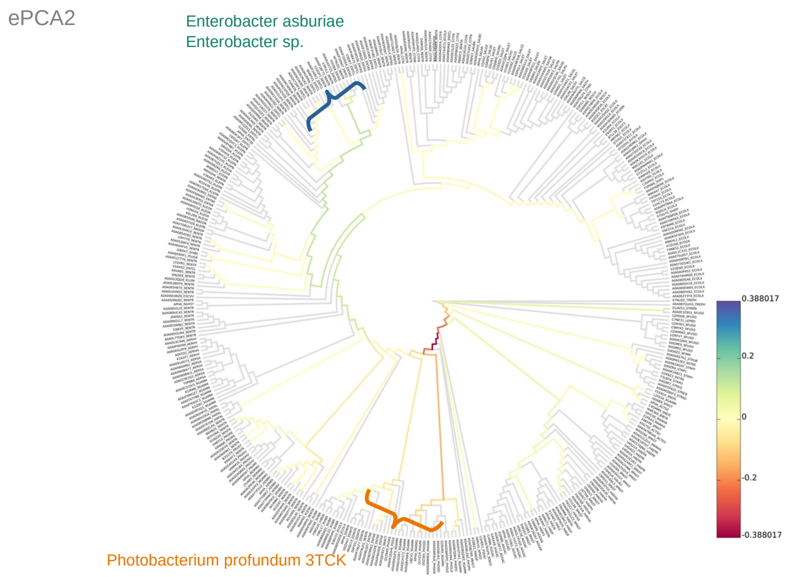

Photobacterium profundum 3TCK

Photobacterium kishitanii

Marinomonas sp. (strain MWYL1)

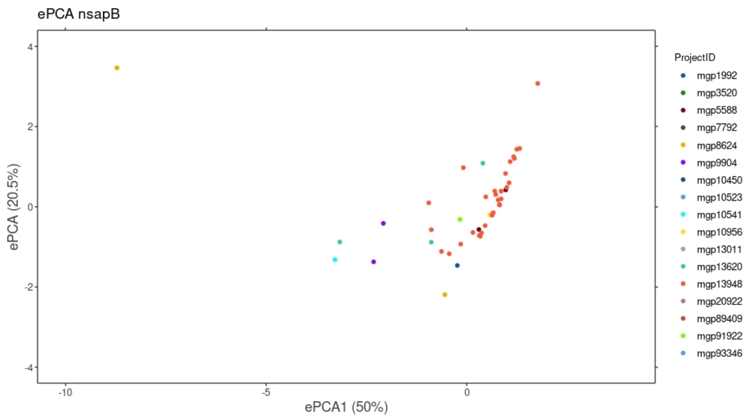

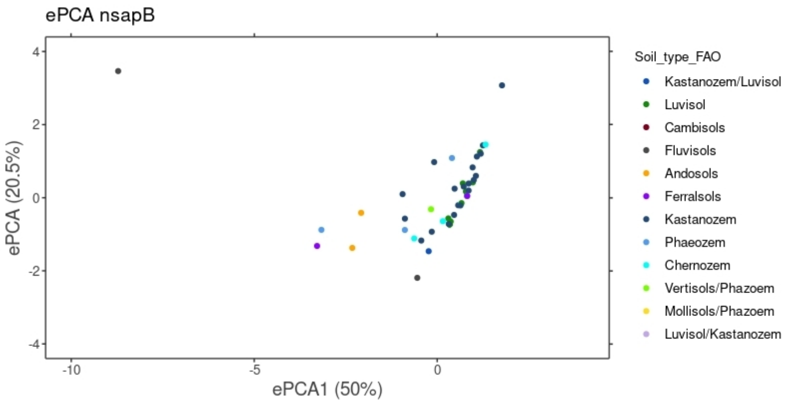

nsapC

ePCA1

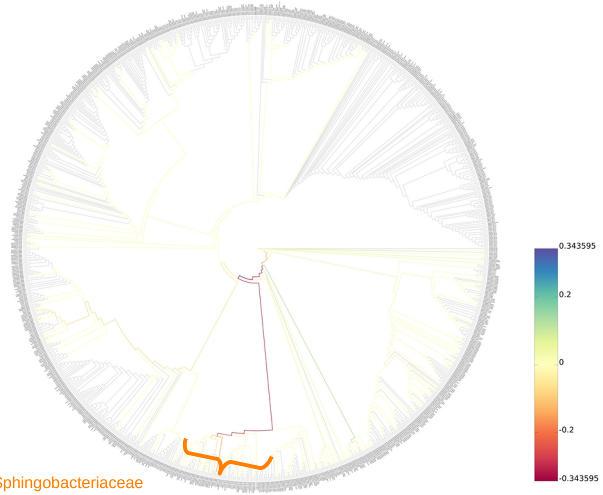

Sphingobacteriaceae

Pedobacter sp. BMA

Mucilaginibacter paludis

Chitinophaga

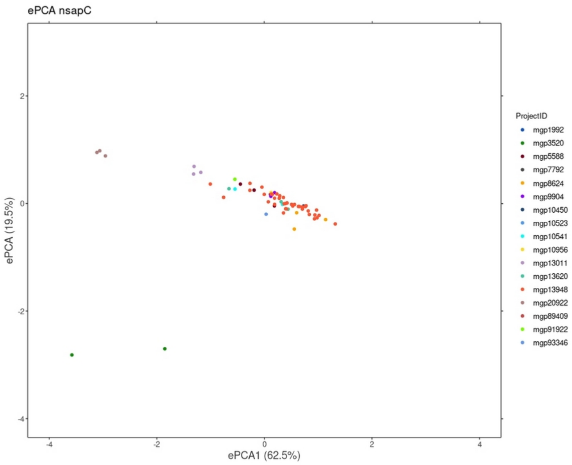

ePCA2

Stenotrophomonas maltophilia

Stenotrophomonas chelatiphaga

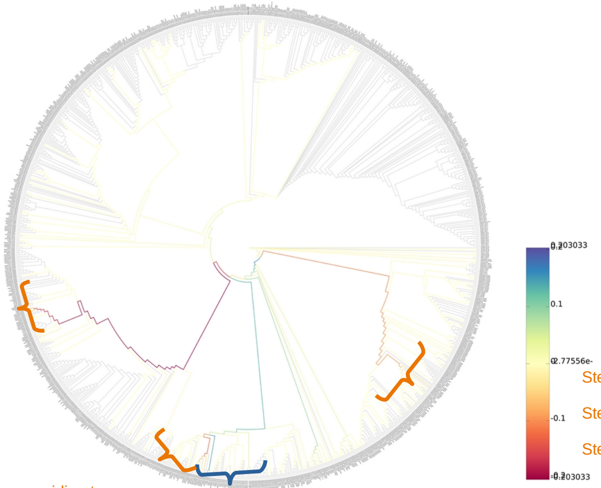

Stenotrophomonas chelatiphaga

Stenotrophomonas maltophilia

Stenotrophomonas chelatiphaga

Pedobacter ginsenosidimutan

Pedobacter sp. Leaf216

Pedobacter kyungheensis

Sphingobacteriaceae

Pedobacter sp. BMA

Mucilaginibacter paludis

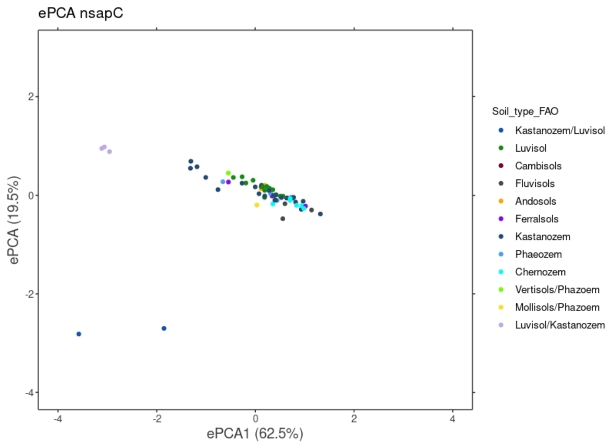

BPP

Cyanothece

Paenibacillus

Bacillus

Bacillus Subtilis

Bacillus Licheniformis

ePCA1

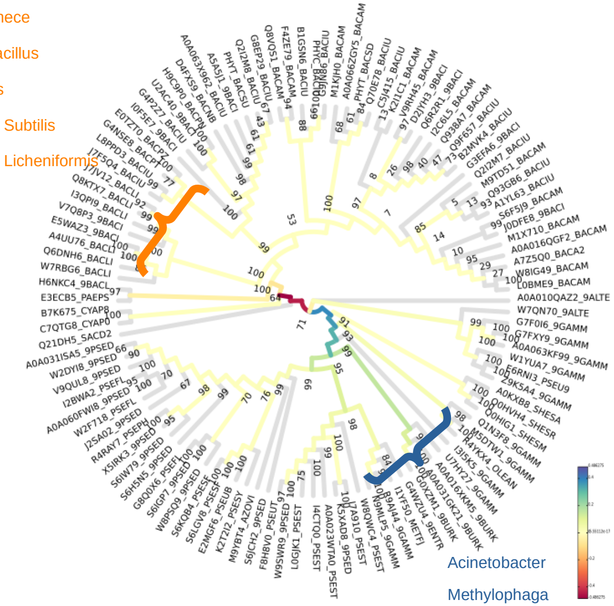

Acinetobacter

Methylophaga

Pseudomonas

Pseudomonas syringae

Shewanella..

Hylemonella

ePCA2

Cyanothece

Paenibacillus

Bacillus

Bacillus Subtilis

Bacillus Licheniformis

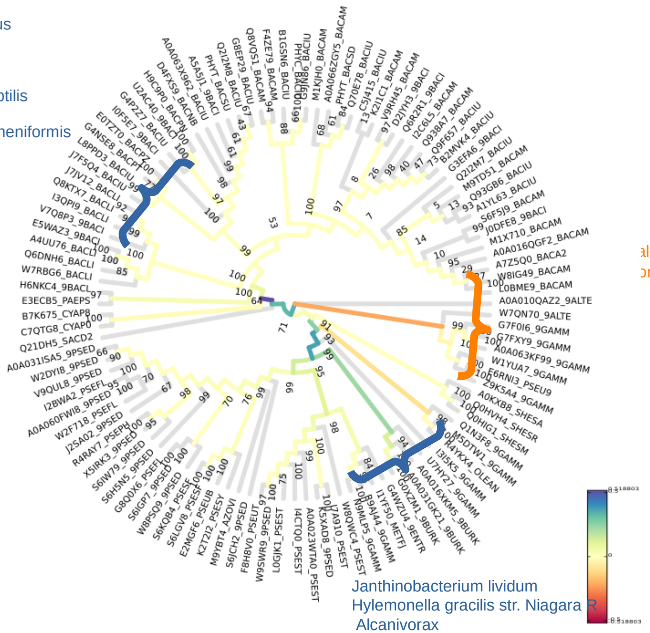

[illegible]alteromonas

[illegible]onadales

Janthinobacterium lividum

Hylemonella gracilis str. Niagara R

Alcanivorax

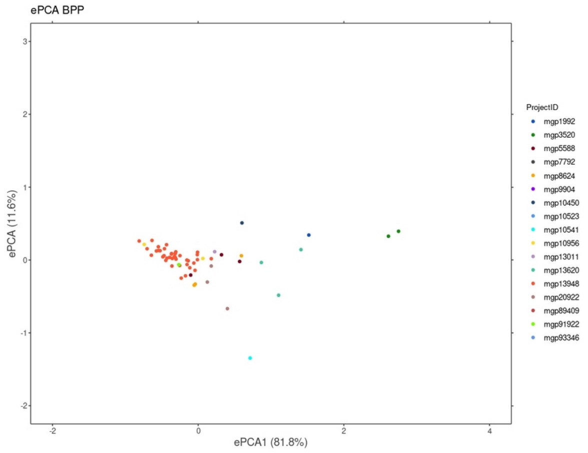

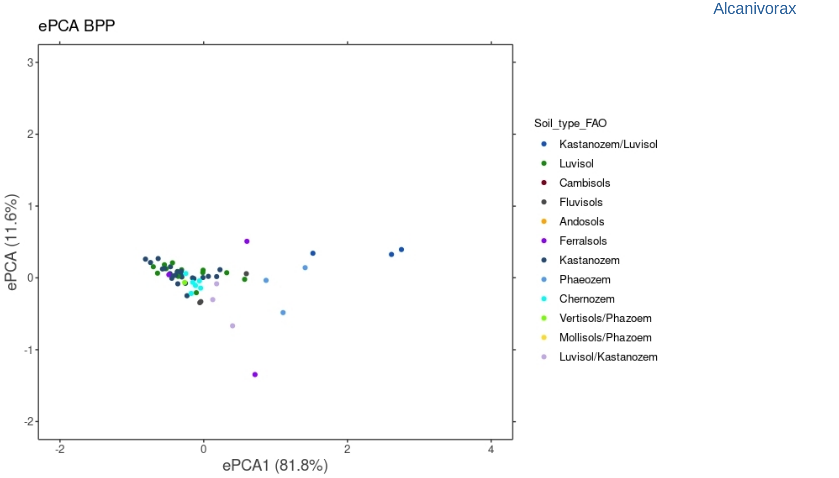

CPhy

Clostridium
Clostridium butyricum
Parachlamydia

Stigmatella aurantiaca
Myxococcus stipitatus Bdellovibrio bacteriovorus str. Tiberius
Acidovorax avenae
Ralstonia solanacearum

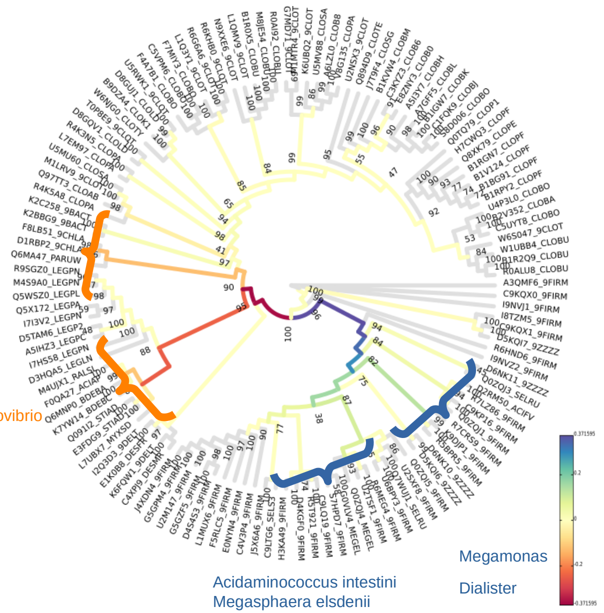

Acidaminococcus intestini
Megasphaera elsdenii
Mitsuokella
Selenomonas ruminantium

Megamonas

Dialister

Selenomonas sp.

Selenomonas sputigena

Clostridium
Clostridium butyricum
Parachlamydia

Stigmatella aurantiaca
Myxococcus stipitatus Bdellovibrio bacteriovorus str. Tiberius
Acidovorax avenae
Ralstonia solanacearum

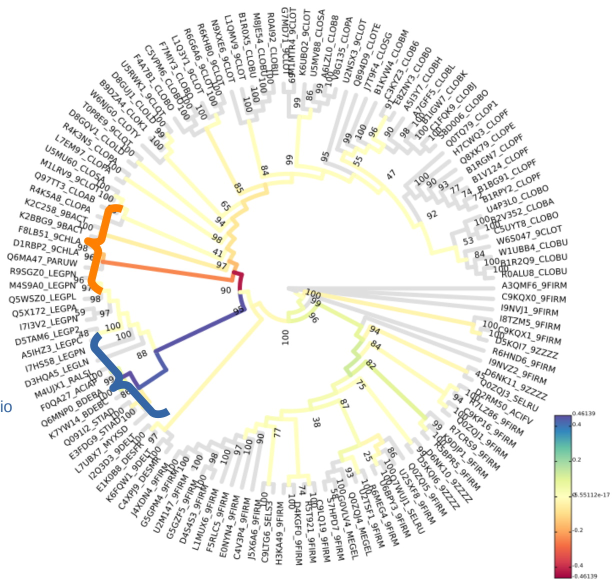

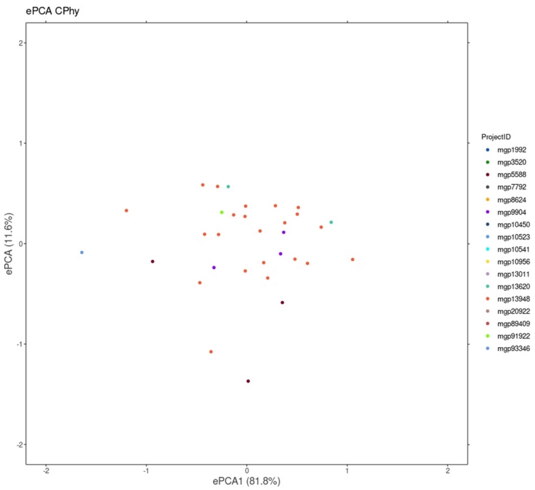

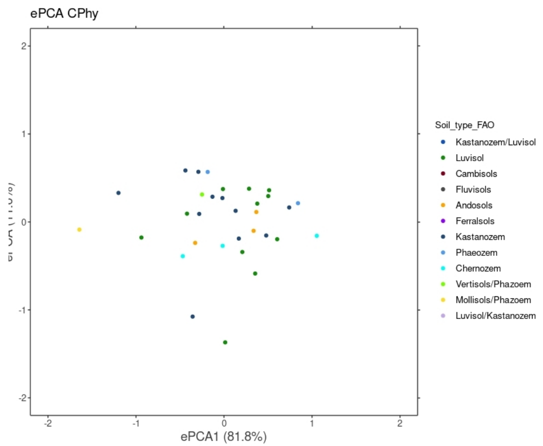

Supplement: Supplementary file 9 — FIGURE S9. Graphic representation of the first two axes of the edge‐PCA for each enzyme using samples as observations. Each point represents samples from the project mpg1992 (blue); mpg3520 (green); mpg5588 (dark red); mpg7792 (gray); mpg8624 (mustard); mgp9904 (violet); mgp10450 (dark blue); mgp10523 (stone blue); mgp10541 (turquoise); mgp10956 (yellow); mgp13011 (lilac); mgp13520 (jade); mpg13948(orange); mpg20922 (brown); mgp89409 (brick‐red); mgp91922 (light green); mgp93346 (light blue). (b) The phylogeny distribution of each enzyme hits along the first and second axis of the analysis (protein with positive coefficients are marked in blue and proteins with negative coefficients are marked in orange). [file EMI4-15-352-s014.pdf]
