# Supplementary material for: Abundance and phylogenetic distribution of eight key enzymes of the phosphorus biogeochemical cycle in grassland soils
Source: Environ Microbiol Rep. 2023 May 10;15(5):352–69. doi: 10.1111/1758-2229.13159 (PMC10472533; doi:10.1111/1758-2229.13159)
Supplement: Supplementary file 6 — FIGURE S6. CAP based on Kantorovich‐Rubinstein distance for non‐specific acid phophatases (Nsap). PERMANOVA analysis with 999 permutations was performed to determine the significance between the sites/project. The variable's vector length represents the correlation between each variable and the axes. For each Nsap were showed two plots, in the first one the samples are colored by project and in the second colored by soil type. [file EMI4-15-352-s018.pdf]

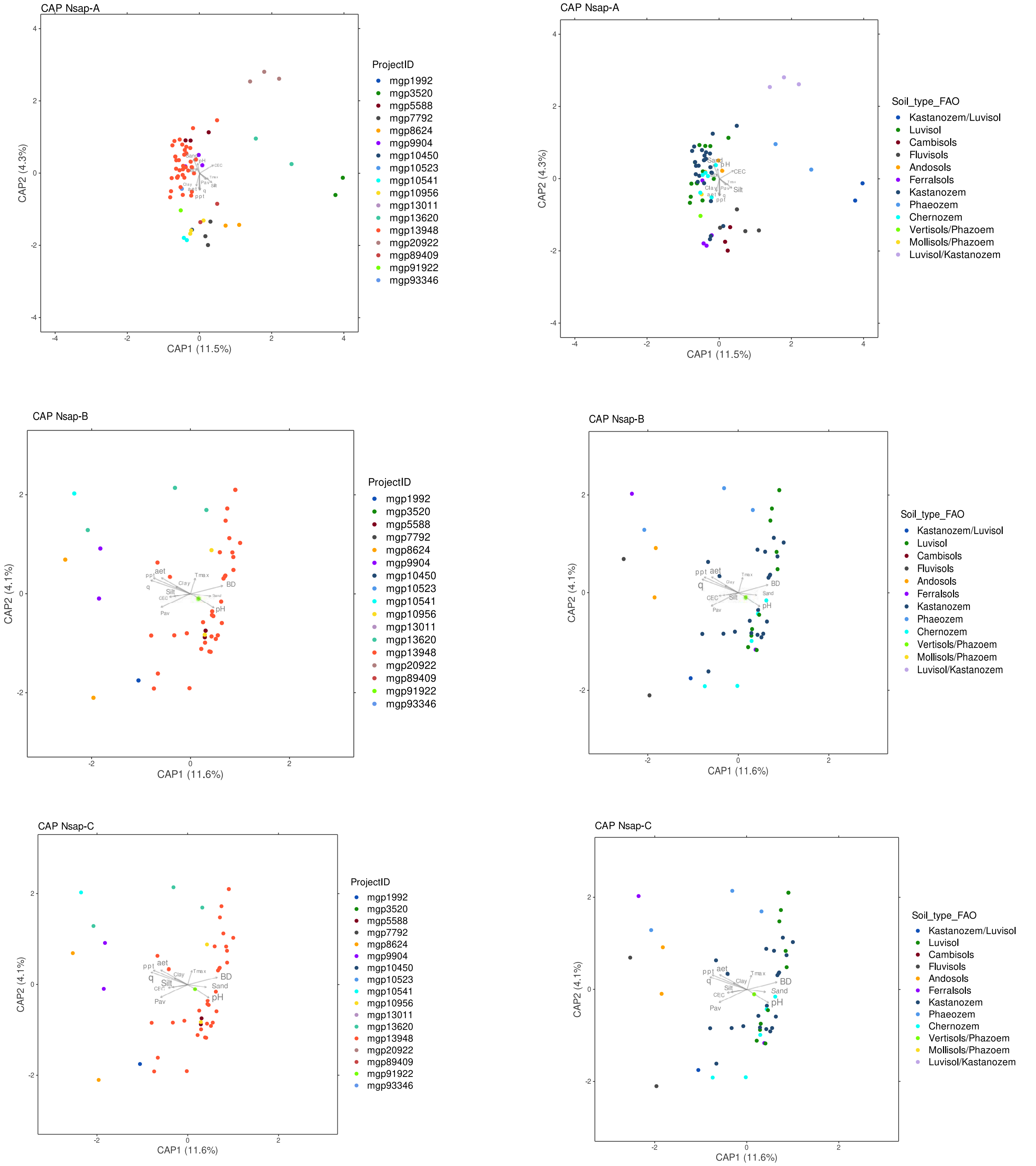
CAP Nsap-A
CAP1 (11.5%)
CAP2 (4.3%)
ProjectID
mgp1992
mgp3520
mgp5588
mgp7792
mgp8624
mgp9904
mgp10450
mgp10523
mgp10541
mgp10956
mgp13011
mgp13620
mgp13948
mgp20922
mgp89409
mgp91922
mgp93346
CAP Nsap-A
CAP1 (11.5%)
CAP2 (4.3%)
Soil_type_FAO
Kastanozem/Luvisol
Luvisol
Cambisols
Fluvisols
Andosols
Ferralsols
Kastanozem
Phaeozem
Chernozem
Vertisols/Phazoem
Mollisols/Phazoem
Luvisol/Kastanozem
CAP Nsap-B
CAP1 (11.6%)
CAP2 (4.1%)
ProjectID
CAP Nsap-B
CAP1 (11.6%)
CAP2 (4.1%)
Soil_type_FAO
CAP Nsap-C
CAP1 (11.6%)
CAP2 (4.1%)
ProjectID
CAP Nsap-C
CAP1 (11.6%)
CAP2 (4.1%)
Soil_type_FAO
ppt
aet
Clay
Tmax
BD
q
Silt
CEC
Sand
Pav
pH
